# 3D Object Recognition Based on Point Clouds in Underwater Environment with Global Descriptors: A Survey

**DOI:** 10.3390/s19204451

**Published:** 2019-10-14

**Authors:** Khadidja Himri, Pere Ridao, Nuno Gracias

**Affiliations:** Underwater Robotics Research Center (CIRS), Computer Vision and Robotics Institute (VICOROB), University of Girona, Parc Científic i Tecnològic UdG C/Pic de Peguera 13, 17003 Girona, Spainngracias@silver.udg.edu (N.G.)

**Keywords:** 3D object recognition, point clouds, global descriptors, laser scanner, underwater environment, pipeline detection, inspection, maintenance and repair, AUV, autonomous manipulation

## Abstract

This paper addresses the problem of object recognition from colorless 3D point clouds in underwater environments. It presents a performance comparison of state-of-the-art global descriptors, which are readily available as open source code. The studied methods are intended to assist Autonomous Underwater Vehicles (AUVs) in performing autonomous interventions in underwater Inspection, Maintenance and Repair (IMR) applications. A set of test objects were chosen as being representative of IMR applications whose shape is typically known a priori. As such, CAD models were used to create virtual views of the objects under realistic conditions of added noise and varying resolution. Extensive experiments were conducted from both virtual scans and from real data collected with an AUV equipped with a fast laser sensor developed in our research centre. The underwater testing was conducted from a moving platform, which can create deformations in the perceived shape of the objects. These effects are considerably more difficult to correct than in above-water counterparts, and therefore may affect the performance of the descriptor. Among other conclusions, the testing we conducted illustrated the importance of matching the resolution of the database scans and test scans, as this significantly impacted the performance of all descriptors except one. This paper contributes to the state-of-the-art as being the first work on the comparison and performance evaluation of methods for underwater object recognition. It is also the first effort using comparison of methods for data acquired with a free floating underwater platform.

## 1. Introduction

The last few years have seen a multitude of object detection and recognition approaches appear in the literature. This development effort has been driven by the growing need to have autonomous systems that can interact with poorly structured, poorly organized and dynamic real-world situations.

Significant progress has been made in object recognition for mobile robots over the last decade. An application scenario that achieved a promising degree of performance is the use of robots in kitchen environments [1,2,3]. Robots are able to identify everyday objects such as bowls, plates and cups using color and depth cameras, in order to locate and grasp them in an automated way. More recently, a new artificial intelligence robotics research lab was opened by NVIDIA where the main focus is to teach a robotic arm to navigate an IKEA kitchen [4] and recognize different utensils. Stereo vision systems were been used for identifying and grasping objects [5,6,7], where the robots aimed to accurately localize parts of the object from images and determine the correct grasping points.

The application of recognition in indoor environments using mobile robots has extended to a wide range of other applications. These include domestic assistance to elderly people or those with a certain degree of disability [8,9,10], agricultural [11,12] and industrial applications [13,14], and in advanced driver-assisted systems [15,16,17,18].

Autonomous driving and indoor service robotics are two main application scenarios which are partially responsible for the surge in work on object detection and recognition. Both scenarios imply robots that operate alongside humans, and whose actions can be potentially dangerous to human life. In this sense, there has been a drive towards increasing both the robustness and speed of the recognition process. For land robotics the increase of robustness can be achieved in part by the use of different complementary sensory modalities, such as laser scanners, Light Detection and Ranging (LIDAR), color cameras, and depth sensors based on texture projection. However, in other application scenarios such as in underwater robotics, the use of complementary sensors may be severely restricted or impossible, due to payload limitations and environmental conditions that are adverse to these types of sensors.

The underwater environment is one of the most challenging in terms of sensing in general and in terms of object perception in particular. The rapid attenuation and scattering of light and other electromagnetic waves implies that object detection and recognition when using optical sensing can only be conducted at very short distances from the objects, in the order of just a few meters. Acoustic propagation allows much longer ranges in terms of sensing distance, but the object representations obtained are far too noisy and coarse in resolution to allow precise object identification and localization for autonomous object grasping. Comparatively fewer applications of object recognition were reported underwater than in the above-water counterpart. These include pipeline identification and inspections based on optical images in seabed survey operations [19], cable identification and pipeline tracking based on acoustic images [20], and recognition of different geometric shapes such as cylinders and cubes [21] using acoustic imaging cameras.

In this paper, we are interested in exploring methods suitable for object recognition underwater, with the future aim of grasping and manipulating such objects. The long-term potential application scenarios are wide ranging, and include:

- Inspection, maintenance and repairing of offshore structures, which are frequently carried out by the oil and gas industries [22].
- Safe and secure exploration of inaccessible, polluting and dangerous maritime resources, including the detection of man-made objects [23,24].
- Subsea collision avoidance, by using systems to identify and locate a different obstacles [25], for example in the early assessment of accident sites.
- Detection and identification of marine wildlife, with the aim of studying their physical environment [26].

### 1.1. Objectives and Contributions

This paper addresses the problem of 3D object recognition in underwater environments. The main goal of this work is to compare the performance of state-of-the-art global methods for the recognition of different man-made objects. It focuses on the use of global descriptors available in the open source library “Point Cloud Library (PCL)” [27].

As elaborated in Section 2, global descriptors have the advantage of better representing the whole object in a compact way, by exploiting relationships between points on different parts of the object. The main drawback of these methods lies in their inability to deal with severe occlusions, and with cluttered data comprising multiple objects. Although local descriptors are more adequate in realistic and clutter scenarios, they have a much higher computation cost. This makes them less suitable for real-time data processing on vehicles with limited computational resources, such as the case of AUVs. Global methods, on the contrary, are more adequate to real-time operation and, for this reason, are the focus of this paper.

The chosen test objects are all related to underwater piping and tubing, and include different types of valves and sections, as detailed in Section 5.1. These objects were selected because they are representative of the building blocks of existing underwater structures where autonomous manipulation is expected to have a high impact in the near future. Given that our primary concern is the recognition of objects whose shape is known a priori, we used Computer Aided Design (CAD) models of the objects in our testing. The CAD models provide a noise-free description of the shape, from which virtual views of the objects can be produced under realistic conditions of added noise and varying resolution.

The results from experiments with real data, collected by an AUV equipped with a fast laser sensor developed in our research centre [28] are used to illustrate how each descriptor works and performs, under realistic subsea conditions. These conditions include, for example, the acquisition of data by a moving platform, which can create deformations in the perceived shape of the objects. Contrarily to aerial laser scanning applications, where the longer imaging range and extra sensing devices (such as GPS) can assist in correcting the effects of the moving platform, in underwater environments these effects are considerably more difficult to correct using the typically available sensors, such as IMUs and DVLs.

This paper contributes to the state-of-the-art as being the first work on the comparison and performance evaluation of methods for underwater object recognition. It is also the first effort using comparison of methods for data acquired with a free floating underwater platform.

Regarding the specific application of pipe-related object recognition, few publications exist in the literature using 3D point clouds as the main (or only) source of information. To the best of our knowledge these are all above-water application scenarios, using high resolution LiDAR. Examples include the work of Huang et al. [13] and Pang et al. [29], where a complex pipeline structure is divided and modelled as a set of interconnecting parts, using a SVM-based approach and a single local feature descriptor (Fast Point Feature Histogram, mentioned in Section 2). Another noteworthy application to pipeline classification is the work of Kumar et al. [30] where an aerial vehicle equipped with a low-cost LiDAR is able to map and identify pipes of different sizes and radii. The pipe identification is based on the analysis of smaller ROIs where information about curvature is gathered. Since the focus of that work is on the real-time mapping, there is no attempt to detect and classify other objects apart from pipes.

### 1.2. Structure of the Paper

The paper is structured as follows. Section 2 presents an overview of object recognition approaches with selected examples of the most relevant previous work. Section 3 provides a deeper description of the class of object recognition methods (Global methods) that are used in this paper, including a description of each of the methods tested. Section 4 explains the algorithmic pipeline used for the processing and testing. Section 5 details the experimental setup. Section 6 provides comparative results obtained first in simulated conditions, and then in a real experiment, using an AUV equipped with an underwater scanner developed at our lab [28]. Finally Section 9 draws the main conclusions and provides future work directions.

## 2. Overview of Object Recognition from Point Clouds

This section presents an overview of the most relevant approaches in the literature related to 3D object recognition from point clouds.

Object recognition approaches can be divided into three broad categories: global, local and hybrid. Global methods aim at representing the whole object as a single vector of values. A definite advantage of these methods is that they are suited for real-time data processing, due to their low computation cost. However, they present the disadvantage of being disturbed by cluttered scenes. To overcome this sensibility to the presence of multiple objects, global methods require a preliminary step of object segmentation, in order to isolate individual objects previous to the recognition. Conversely, the local methods are generally more specific for a local area and computed from salient points, which make them more robust to clutter and occlusion. These methods seek to describe the object as a collection of small salient areas of the object, whose geometric arrangement is also taken into account. However, these methods suffer from larger computation cost due to the large number of points-descriptors per object. The last category is hybrid methods, which aim at incorporating the strengths of both global and local descriptors.

### 2.1. Local Recognition Pipelines

The use of local descriptors for 3D object recognition was reported in several review papers.

Alexandere et al. [31] assessed the different descriptors implemented in the PCL, considering only the methods that could be applied directly on a point cloud. As such, some methods were excluded, namely the Spin Image Descriptor [32] which is based on a mesh representation, the Global Fast Point Feature Histogram (GFPFH) [33] which assumes labelling of the points, and the Camera Roll Histogram [34] given that they were mainly interested in evaluating the recognition process without estimating the pose of the objects. The tests were carried out based on an RGB-D object dataset [35]. The authors singled out the Colored Signature of Histogram of Orientation (CSHOT) descriptor [36] given that it offered a good balance between recognition performance and time complexity.

A comprehensive survey paper by Guo et al. [37] reported and reviewed the most important local descriptors applied on mesh surface or point clouds. These authors considered the existing local descriptors published between 2001 and 2015. The local descriptors presented in [37,38] were tested on four relevant benchmark datasets: Bologna [39], the UWA 3D Object Recognition (U3OR) [40], the Queen’s [41] and the Ca’ Foscari Venezia Dataset [42].

For details of 3D local feature descriptors, we refer the reader to [37,38]. In the following, we provide a brief review of several local and especially global descriptors based on point clouds that relate to our work.

After 2015, the literature continues and includes other local descriptors. For instance, the Equivalent Circumference Surface Angle Descriptor (ECSAD) [43] is a 3D shape feature designed for detecting the 3D shape edges, and is best suited when the objects have clear prominent edges. Another local descriptor based on contour information is the Rotational Contour Signatures (RCS), presented in [44]. The RCS computes several signatures from 2D contour information, obtained from 3D-to-2D projection of the local surface. The key contribution of these authors consisted in building a geometry encoding, where the local surface is rotated toward a predefined local reference frame, thus enabling the gathering of multi-view contour information. The RCS descriptor was compared against five state-of-the-art descriptors, including Spin Image (SI) [32], SNAPSHOTS [45], Fast Point Feature Histograms (FPFH) [46], SHOT [47] and Rotational Projection Statistics (RoPS) [48] and using the two standard databases: the Bologna [39] and the UWA Object Recognition (UWAOR) datasets [40].

Recently, using Mobile Laser Scanning (MLS) point cloud data, Zhenwei et al. [49] classified pole-like objects from unstructured MLS point cloud data. The authors used the random sample consensus (RANSAC) [50] and principal component analysis (PCA) [51] to detect the vertical cylinder model and principle direction of the point set. Along the same line, a good description of the state-of-the-art for mobile laser scanning systems is presented in [52]. The authors cite several methods based on the point cloud data used for gathering information on road and transport, with emphasis on relevant methods for feature extraction, segmentation and object detection.

### 2.2. Global Recognition Pipelines

Global descriptors describe the characteristics of the entire object and they are often used as a coarse representation suitable for real-time applications. Most of the existing global descriptors evolved from local feature representations. An example of this is the Viewpoint Feature Histogram (VFH) [53], which is an extension of the local descriptor Fast Point Feature Histograms (FPFH) [46], that encodes information on the whole object and viewpoint. Rusu et al. [53] validated the efficiency of the VFH applied on 60 IKEA kitchenware objects collected using stereo cameras. The method was compared against the state-of-the-art Spin Image (SI) [32] with favorable results for VFH. This descriptor is designed to accomplish both recognition and pose identification.

The Global Radius-based Surface Descriptor (GRSD) [54] is a global version of the local Radius-based Surface Descriptor (RSD) [55] suitable to mobile manipulation applications. To evaluate their approach, numerous experiments were performed in an indoor environment. These experiments rely on geometric and appearance-based data, where everyday objects were used. The recognition approach used both images collected with a stereo camera and 3D depth data from a range scanner. Marton et al. [54] proposed an approach aimed at combining the Speeded-up Robust Features (SURF) [56] 2D descriptor with the GRSD 3D descriptor. The authors defined a hierarchical classification system, where the GRSD is used as a first step to reduce the number of choice of objects to those of similar shape, followed by the use of the SURF descriptor to accurately identify the object in a particular instance. Gunji et al. [57] proposed the Bag-of-Features (BoF)-based object recognition pipeline, which is suited to processing large-scale scene point clouds acquired in indoor environments with a laser rangefinder. This method follows a two-step approach. The first step is a preprossessing of data which includes an unsupervised training of codebook (collection of vector-quantized features) using K-means, where the codebook consists of centroids of clusters of FPFH, a local descriptor. The second step is the recognition of the target model, which is implemented by computing the BoF inside a sliding window. The authors performed trials using real data, where they showed that the proposed approach based on BoF has better performance in terms of precision and recall compared to the 3D Hough voting [58] with SHOT descriptor [47] method. Similar work based on Bag-Of-Feature was proposed in [59].

Jain et al. [60] introduced another global descriptor derived from a local descriptor. The authors presented a manipulation framework for grasping objects based on Global Principal Curvature Shape Descriptor (GPCSD). The GPCSD aimed to categorize object-clusters that are geometrically equivalent into similar primitive shape categories. The GPCSD is based on the local Principal Curvatures (PC) values. The computation of the descriptor is similar to GRSD presented in [54]. However, rather than labelling the voxel using Radius-Based Surface Descriptor (RSD), the authors applied Principal Curvatures (PC). The performance of GPCSD is compared against Global Radius-Based Surface Descriptor (GRSD), using the Washington RGB-D dataset and real-time data from a Microsoft Kinect. The results showed that both descriptors performed well, although GRSD was found to be more robust to distance variations.

### 2.3. Hybrid Recognition Pipelines

From the results of the studies above, it is natural to expect a better performance by merging global and local information. The following reports present different hybrid recognition pipelines that combine both approaches.

In [61], Aldoma et al. presented a hybrid pipeline allowing the processing of data from different modalities. The method is based on three different descriptors: the SIFT 2D local descriptor, the 3D global descriptor OUR-CVFH descriptor [62], that exploits the color, shape, and object size information, and the SHOT [47] descriptor, a 3D local descriptor. The two local (2D and 3D) and the 3D global descriptors were combined using an optimization-based hypothesis-verification method, which aimed at validating a subset of hypotheses belonging to recognition hypotheses.

Alhamzi et al. [63] used state-of-the-art 3D descriptors to recognize the objects and estimate their pose, using the PCL Library. The authors selected the Viewpoint Feature Histogram (VFH) [53], a global descriptor, to recognize the objects of interest, whereas the Fast Point Feature Histogram (FPFH) [46], a local descriptor, was applied to estimate the position of the object. The performance of VFH was compared to the state-of-the-art descriptors, namely the Ensemble of Shape Functions (ESF) [64] and the Clustered Viewpoint Feature Histogram (CVFH) [34]. Then the authors integrate the result of the VFH descriptor, with five various types of local descriptors: SHOT [47], CSHOT [36], PFH [65] PFHRGB [27] and FPFH [46]. Alhamzi et al. concluding that the couple VFH and FPFH achieved the best result. The performance of the hybrid method-based VFH and FPFH was validated using the Willow Garage dataset [66].

Sels et al. [67] presented a new fully automated Laser Doppler Vibrometer (LDV) measurement technique. Their measurement technique was remarkable in using data from a 3D Time-of-Flight camera jointly with a CAD file of the test object to automatically obtain measurements at predefined locations. The authors adopted the same pipeline presented in [63], where the global VFH descriptor was used for recognition, and the local FPFH descriptor to estimate the pose of the object.

## 3. Global Descriptors

As mentioned before, the central idea behind the methods for object recognition from 3D points is that an object can be characterized by a set of combined features, either local or global. This section presents a more detailed summary of the class of methods that are used in this paper: global descriptors. All the approaches that are tested and compared in the results section are here described.

The global features describe and encode the shape or geometry information of the object in a very compact way, allowing a low computational effort. The local features represent the objects by encoding subsets of neighbouring points around each salient point, which implies a much larger dimension of the feature space. However, the local descriptors have the advantage of dealing with high object cluttering and occlusions.

The data used in the underwater experiments of this paper was collected using a laser scanner developed in-house, that generates point cloud data without color information. Acquiring reliable color information underwater is quite a challenging task, due to the absorption and attenuation which are strongly dependant on distance and on the light wavelength. As such, from the robustness point of view, it is important to develop and use methods that do not rely on color. A set of descriptors were therefore selected which do not require color information and are available in the Point-Cloud Library. The only exception is the Global Orthographic Object Descriptor (GOOD) that is not integrated in the current version 1.8 of PCL [27].

In this study, the evaluation of a set of global descriptors is performed taking into account their performance, whether they retain their descriptiveness under flexible transformations based on how the object was scanned, under variations in the density of point clouds, and under different levels of noise. These descriptors are represented by a histogram, whose the size depends on the descriptors themselves.

The recent literature shows a gradually increasing interest in using methods available in the Point Cloud Library (PCL library). The methods evaluated and compared in our study were considered to be the most relevant in the literature, and are shown in Table 1, and include the Viewpoint Feature Histogram (VFH) [53], the Clustered Viewpoint Feature Histogram (CVFH) [34], the Oriented, Unique and Repeatable CVFH (OUR-CVFH) [62], the Global Orthographic Object Descriptor (GOOD) [68], the Ensemble of Shape Functions (ESF) [64], the Global Fast Point Feature Histogram (GFPFH) [33] and the Global Radius-based Surface Descriptors(GRSD) [54]. The list is ordered by chronological descending order of the methods they are based on.

In the following subsections, the 3D global descriptors of the study are briefly introduced.

### 3.1. Global Orthographic Object Descriptor (GOOD)

The Global Orthographic Object Descriptor (GOOD) [68] aims at providing reliable information in real time. To boost the robustness, a unique and repeatable object reference frame was applied. When computing the local reference frame, a sign ambiguity arises, which is solved with a proposed method based on eigenvalues and Principal Component Analysis (PCA). Using this reference frame, three principal orthographic projections are created (XoZ, XoY, and YoZ). Each orthographic projection is partitioned into bins, where the number of points falling into each bin is counted. The authors performed several tests, changing the number of bins, to find an adequate bin size that achieves best performance. These bins were presented as distribution matrices. The descriptor is finally obtained by concatenating these distribution matrices, where the sequence of projection was determined based on the highest entropy and variance of the projections. The size of GOOD histogram equal 75 floats, 25 per each one of the matrix of distribution of the three projections.

**Figure 1:**
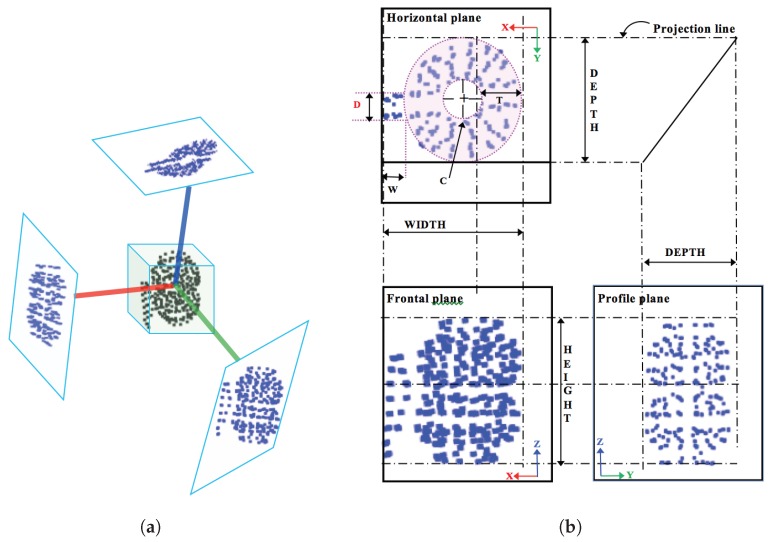
Example of how the three orthogonal projections are built: (**a**) Local reference frame and projections; (**b**) Projections in multi-view layout. GOOD can also be used for object manipulation. In the plan view of the object, the symbols C, W, D and T represent how the projection can be further processed where the features for the manipulation task can be extracted, namely inner radius (C), thickness (T), handle length (W) and handle thickness (D).

The crucial advantage of GOOD, is the fact that it is represented by three orthographical projections, which make it rich in terms of information suited for manipulation tasks. As illustrated in Figure 1, it is essential to know the true dimensions of the object in order to adjust the gripper, and this information can be obtained from the dimension on orthographic projection grid.

### 3.2. The Ensemble of Shape Functions (ESF)

**Figure 2:**
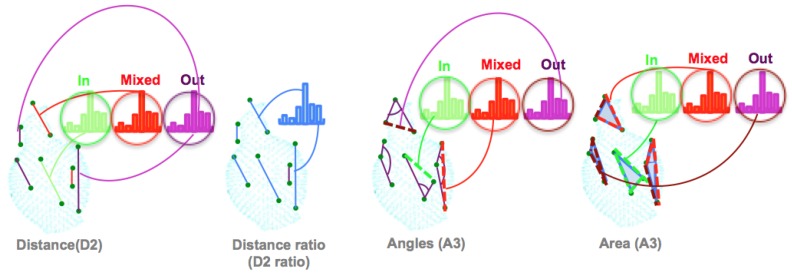
The Ensemble of Shape Function (ESF). Illustration of how shape functions are computed for a point cloud of an amphora. Left: point distance distributions. The green histogram represents points in, the red histogram points out, and the purple represents mixed points; Middle left: Distance Ratio; Middle right: angle distributions; Right: the area covered by triplets of sampled points.

The Ensemble of shape functions (ESF) was introduced by Osada et al. [69]. The authors suggested a way to characterize any 3D polygonal model, using a geometric shape function based on five measurements. Later on, Wohlkinger et al. [64] used the same principle, but reduced the number of measurements from five to three. The ESF descriptor combines a set of ten 64-bin-sized histograms of shape functions, describing geometric properties of the point cloud. The descriptor uses a voxel grid to approximate a real surface. Then, for each point in the cloud, three points are chosen randomly. These points are used to compute the three shape functions, as illustrated in Figure 2:

- The distance D2: This is the distance calculated between two points, then classified into one of three categories based on whether the connection line falls in the surface, off the surface, or is mixed (with one part in and the other off the surface). To characterize the distribution of the voxels along the line, the authors added the ratio of line distance (D2 ratio). This ratio is equal to zero if the line falls off the surface, equal to one if inside, and equal to the numbers of the voxels, along with the connection, if the line is mixed.
- The angle A3: This is the angle computed between two lines, then the line opposite to this angle is classified in one of the three categories (in, out, or mixed).
- The area D3: This is the square root of the area formed by the three points, based on the Heron Formula (1).

(1) $$\begin{array}{cc} D3 & =\sqrt{\sqrt{s(s-a)(s-b)(s-c)}},\\ s & =\frac{a+b+c}{2}, \end{array}$$

where *s* represents the semi-perimeter and *a*, *b* and *c* the side lengths of the triangle. The area is classified similarly as in D2.

The total length of the ESF descriptor is 640 bins which are divided into ten sections: three for the angle component (A3), three for the area component (D3), three for the distance component (D2) and one for the ratio of distance component (D2 ratio).

### 3.3. Global Radius-Based Surface Descriptors (GRSD)

The Global Radius-based Surface Descriptor (GRSD) was introduced by Marton et al. [54]. The descriptor is considered as the global version of the Radius-based Surface Descriptor (RSD) [54] which is a local descriptor.

To better understand the GRSD, we start by describing how the RSD is computed. The RSD descriptor encodes the radial relationship between every pair of points lying in a surface (defined by a radius *r*). For each query point *p* and its neighbour points $p_{i}$, the distance and angle α formed between the two normals of the pair *p* and $p_{i}$ are computed. We could draw an imaginary sphere around the pairs of points, where the point *p* belongs to each of these spheres. From all the possible cases, only points with the largest and smallest spheres are chosen, and their radii are selected to build a descriptor of the point $radius=\left[r_{min},r_{max}\right]$.

(2) $$\begin{array}{cc} d & =\sqrt{2r}\sqrt{1-\cos(\alpha )},\\ r & \approx \frac{d}{\alpha }, \end{array}$$

To compute the GRSD descriptor, first, the input point cloud is voxelized. Once the neighborhood is defined based on the current and surrounding voxels, the RSD descriptor is computed as explained above. Based on the estimation of the two principal radii $r_{min}$ and $r_{max}$, the surfaces are categorized based on intuitive rules defined in [54]. These surfaces are classified into: planes (large ($r_{min}$), cylinders (medium $r_{min}$, large $r_{max}$), edges (small $r_{min}$, $r_{max}$), rims (small $r_{min}$, medium to large $r_{max}$), and spheres (similar $r_{min}$, and $r_{max}$). Once all voxels are categorized locally into one of these classes, the GRSD histogram is binned based on the number of transitions between all these local labels. The GRSD descriptor labels these transitions between distinctive surface types for an object.

(3) $$\begin{array}{cc} b & =\frac{s\cdot (s+1)}{2} \end{array}$$

where *s* is the number of possible categories, resulting in 21 dimensions for these 6 possible categories.

### 3.4. Global Descriptors Based on Fast Point Feature Histogram (FPFH)

This section outlines the family of global descriptors based on the computation of the local descriptor Fast Point Feature Histogram (FPFH) [46].

#### 3.4.1. Viewpoint Feature Histogram (VFH)

**Figure 3:**
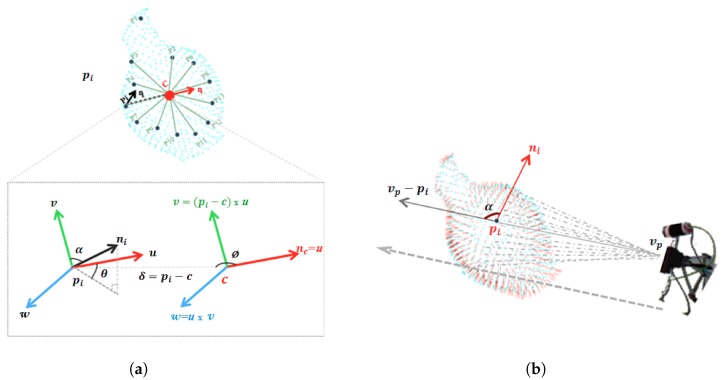
The two components of VFH: (**a**) Extended Fast Point Feature Histogram (EFPFH), the Computation of the three angular features. (**b**) the Viewpoint histogram. The histogram contains the stack of the angle *α* formed by the viewpoint toward the point *p_i_* and its normal.

The VFH was introduced by [53] as a global version of both the Point Feature Histogram (PFH) and the Fast Point Feature Histogram (FPFH) [46]. VFH describes the whole point cloud while PFH/FPFH are based on describing the local geometry around the key-points. The VFH has two components:

1. The Extended Fast Point Feature Histogram (EFPFH). This is an extended version of the FPFH. The difference between EFPFH and its predecessors lies in the way the geometry characteristic of the features is computed. For each point inside the point cloud, instead of comparing each couple of points inside predefined radii, the EFPFH compares each point with the centroid of the point cloud. The histogram is computed using the following steps, where the object is assumed as being a single cluster of points.
  - The centroid of the point cloud (*c*) is computed together with a normal ($n_{c}$).
  - For each point $p_{i}$ and its normal $n_{i}$ in the cluster, a reference frame is defined for each pair $p_{i}$ and *c*, with origin in *c*, where the 3 axes of the frame are $u=n_{c}$, $v=(p_{i}-c)\times u$, and w=u×v.
  - As illustrated in Figure 3a. From *c*, $p_{i}$, $n_{c}$ and $n_{i}$, a set of features are computed from 3 angles (α, θ, ϕ) and the distance γ, as:
    (4) $$\begin{array}{cc} EFPFH & =\left\{\begin{array}{cc} \alpha & =\arccos(v\cdot n),\\ \phi & =\arccos(u\cdot \frac{pi-c}{∥pi-c∥}),\\ \theta & =\mathrm{atan}2\left(w\cdot n,u\cdot n\right),\\ \gamma & =∥pi-c∥ \end{array}\right. \end{array}$$
2. The viewpoint histogram. It is a histogram of the angles between the two vectors as shown in Figure 3b; the vector $v_{p}-p_{i}$ formed from the point $p_{i}$ to the viewpoint $v_{p}$, and the normal $n_{i}$ of the point $p_{i}$.

The complete size of the VFH descriptor is 308 bins composed of The EFPFH; 45 bins for each one (α, θ, ϕ, γ), plus 128 bins for the viewpoint component.

#### 3.4.2. Global Fast Point Feature Histogram (GFPFH)

The Global Fast Point Feature Histogram (GFPFH) [33] is an extended version of the Fast Point Feature Histogram (FPFH) local descriptor. It is based on computing the number of angle histograms between angles of normals of each surface point and its neighboring points as explained in the Section 3.4.1. However, instead of comparing each point with the centroid of the point cloud, FPFH compares each pair of points $p_{i}$ and $p_{j}$ inside predefined radii, considering only the pairs with their direct neighbors.

The GFPFH descriptor needs a preliminary step, which consists of categorizing the surface into classes. These classes depend on the object and how it can be handled or decomposed for grasping. As an example, a cup is composed of a cylindrical body and handle where it can be grasped. Then, for each point, the FPFH is computed. In [33] the authors used The Conditional Random Field model [70] to label each surface with one of the object-classes.

Using the categorization results, the GFPFH descriptor is computed. The first step consists of representing the input point clouds by an octree, where each leaf contains a set of points. For each leaf, a probability of belonging to a particular class is assigned. This probability is computed as the ratio of the number of points in the labeled leaf according to that class over the total number of points.

**Figure 4:**
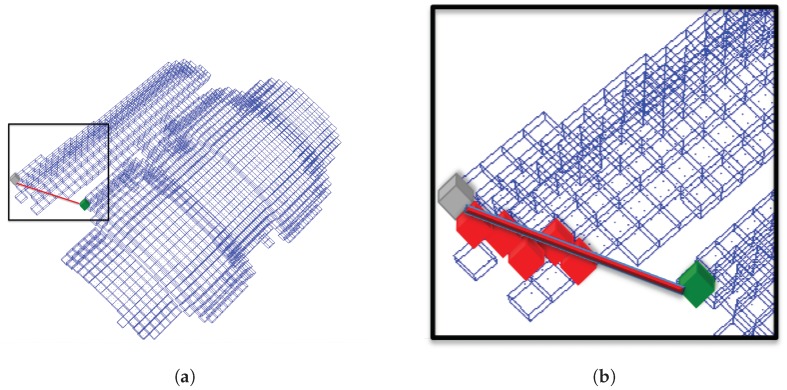
The estimation of a GFPFH for a 3D point cluster. (**a**) Octree representation of the point clouds. (**b**) illustrated zoom in of the region marked in (**a**), for every two pairs of leaves, a ray is cast from the start leaf (green) to the goal one (grey). All intersections with other leaves and free space are recorded and ported into a leaf class pair histogram.

In the following step, a line segment is created as illustrated in Figure 4, where the intersected leaf in its path is checked to see if it is occupied. The results are stored in a histogram based on the leaf occupancy: 0 if it is empty and, the leaf probabilities if it is occupied.

#### 3.4.3. Clustered Viewpoint Feature Histogram (CVFH)

The CVFH global descriptor was proposed by Aldoma et al. in [34] as an extension version of Viewpoint Feature Histogram. It aimed at solving the major limitations in VFH, that were essentially sensitivity to noise and occlusions (where the object has missing parts).

To make CVFH robust against occlusion, the authors proposed discarding the histogram normalization step used in computation of VFH, allowing the CVFH to become scale independent. To be able to distinguish objects with identical size and normals distribution, the authors added a Shape Distribution Component (SDC) in the computation of the histogram. The SDC encodes information about the distribution of the points $p_{i}$ around the centroid of the region measured by the distances:

(5) $$SDC=\frac{{\left(c-p_{i}\right)}^{2}}{max{\left(\left(c-p_{i}\right)\right)}^{2}}$$

where i=1,2,⋯,N, *c* denotes the centroid of the whole surface points and *N* represents the total number of the whole object surface points.

Rather than computing a single VFH histogram for the entire cluster, the main idea of the CVFH consists in splitting the object into stable regions by using smooth region growing segmentation algorithm [63]. For each region a VFH descriptor is computed.

The main advantages of the descriptor compared to its predecessor comes from the decomposition into a set of descriptors of the set of VFH clusters, which represents a multivariate description of the partial view. As long as any of the stable regions is visible, occlusions can be handled. The size of the CVFH is equal to the size of the VFH, where the number of bins used for this component is again 45 thus making a total size of 308 for CVFH.

#### 3.4.4. Oriented, Unique and Repeatable CVFH (OUR-CVFH)

Despite the good result obtained in 3D recognition using CVFH [34], this descriptor suffers from two major drawbacks. On one hand, there is an absence of an aligned Euclidean space, causing the feature to lack a proper spatial description. On the other hand, it is invariant to rotations around the roll of the camera axis, thus restricting the pose estimation to 5 DoF.

In [62] Aldoma et al. presented the oriented, unique and repeatable CVFH (OUR-CVFH) descriptor, the last extension of FPFH. OUR-CVFH descriptor used semi-global unique and repeatable reference frames (SGURF) on object surfaces. The objective of using SGURF is to overcome the limitations of CVFH by defining multiple repeatable coordinate systems on the surface *S*.

## 4. Object Recognition Pipeline

**Figure 5:**
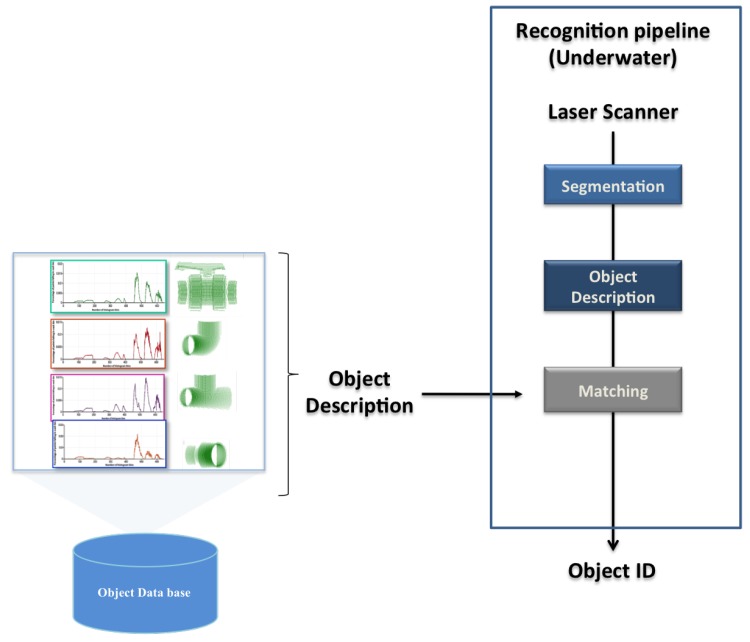
Block diagram of the proposed method.

To compare the descriptors, a 3D object recognition pipeline was used, which is described hereafter. Its block diagram appears in Figure 5. The pipeline is fed with an input scan coming either from a laser scanner (real experiments) or a virtual 3D camera (simulation). Then, a three step process is followed.

1. Segmentation: Real scans pass through a segmentation phase, to remove any point not belonging to the object view. For instance, if the object is lying on the bottom of a water tank, removing the principal plane (the bottom) is enough to correctly segment it. This is actually how it was implemented for the real experiments reported in Section 7. This step is skipped in the simulated results. The proposed 3D recognition pipeline requires a segmentation step, which aims to separate the 3D points belonging to the objects of interest from the rest of the scene. It consists of regrouping the points representing the object into one homogeneous group based on similar characteristics following the approach proposed in [71].
2. Description: This block uses the global descriptors, presented in the previous section, to encode the segmented object (input scan) in a compact way. The global object descriptors are also used to encode the object views stored in the database (object model). In this way the segmented input scan can be matched against the object model views in the database.
3. Matching: This step compares the segmented input scan with all the views of the object models in the database. The matching stage is based on computing Chi-square distance as proposed in [65,72]. The selected view corresponds to the one with the minimum distance.

The output of the recognition module is the object ID of the recognized object, as well as its matching view.

## 5. Experimental Setup

The main goal of this paper is to compare the robustness and performance of the global descriptors and to select the most adequate one for object recognition in an industrial underwater environment. A series of experiments were conducted in order to study the capabilities of the descriptors to distinguish among objects commonly present in Inspection Maintenance and repair (IMR) applications. The proposed objects database was composed of seven objects (Table 2) which are representative of an industrial scenario. The influence of the following parameters in the object recognition capabilities were studied:

- The use of full vs.partial views.
- The point cloud resolution.
- The presence of noise.

Two types of experiments were performed:

1. **Simulated Experiments,** which involved the use of a virtual camera to generate a simulated point cloud of the object, grabbed from a random point of view. The virtual scan was characterized with all the descriptors being used to recognize the object. For each *<object, resolution, noise, full/partial view>* combination, n=100 Montecarlo runs of the experiment were performed, computing the average object recognition, and the confusion matrix.
2. **Real Experiments** involved the use of a laser scanner mounted on the GIRONA 500 AUV operating in a water tank scenario. Four objects were placed on the bottom of the water tank. The GIRONA500 vehicle was tele-operated to follow an approximated square trajectory, starting from a position where the reducing socket was within the field of view of the laser scanner. The trajectory ended with the robot on the ball valve, after passing over the elbow and reducing tee. The vehicle performed 3 complete loops, allowing it to acquire multiple views of the same object, each time it passed above it. The laser scanner was mounted looking towards the bottom providing full views of the objects.

### 5.1. Object Database

The experiments were conducted using the objects illustrated in Table 2. Each one was modelled as a complete set of potentially overlapping views stored as point clouds, covering the full object. The views were virtually scanned from the 3D CAD model using a method similar to the one reported in [34].

### 5.2. Virtual Scan

**Figure 6:**
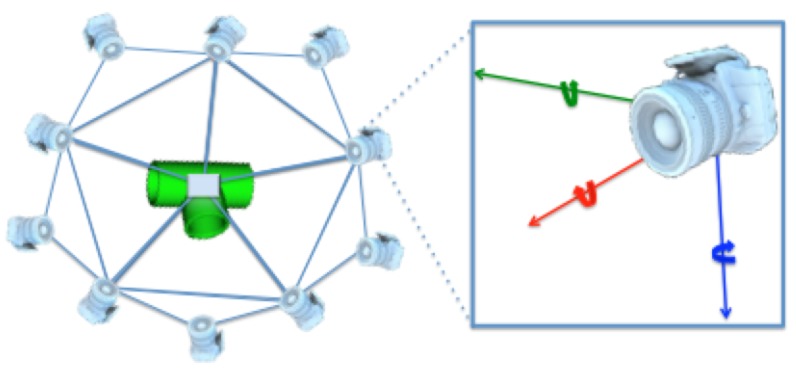
View points used for each object model stored in the database.

A simulated point cloud is generated using the tessellated sphere module from the PCL library [27]. The 3D CAD model of the object is placed at the origin of 3D space. Next, a sphere with a radius equal to the intended camera-to-object distance is used. The sphere is converted into a polyhedron depending on the level of tessellation, as illustrated in Figure 6. The virtual camera is then placed at each corner of the polyhedron. The number of views acquired is therefore equal to the number of corners (Equation (6)).

(6) $$g\left(l\right)=\left(\;4^{l}\times 20\right)/2+2\;;\;\mathrm{being}\;l\;\mathrm{the}\;\mathrm{level}\;\mathrm{of}\;\mathrm{tessellation}$$

The two types of object views were used:

- **Full Object Views** using a level of tessellation fixed to 1, resulting in an icosahedron composed of 20 triangles and in 12 corners. The virtual camera was placed at each corner, at 0.5 m distance looking towards the origin, resulting in 12 full object views (Figure 6). These are the type of views used to represent the object in the database.
- **Partial Object Views** using a random vertex of the icosahedron to place the camera. The camera-to-object distance is randomly selected within the 0.2 to 1 m interval which is representative of the typical range for manipulation operations. The camera is also rotated around the three axes with a random angle of up to ±10°.

### 5.3. Resolution

The XY resolution of the virtual 3D camera used to grab the point cloud was set at 150×150 pixels providing a dense point cloud at the working distances. Nevertheless, the point cloud was sub-sampled at different voxel sizes (Table 3) to study the influence of the scan resolution in the object recognition results.

## 6. Results on Simulated Data

**Figure 7:**
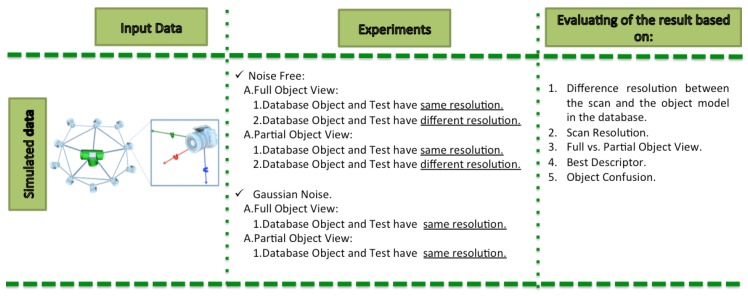
Diagram of the proposed simulated experiment.

The diagram below summarizes the experimental approach followed in this study as shown in the experiments column (Figure 7). The last column indicates a set of criteria that was used for the structuring the performance comparisons and the interpretation of the results. These criteria are the following: Difference of resolution between the scan and the object model in the database, Scan Resolution, Full vs. Partial Object View, Best Descriptor and Object Confusion.

Four different experiments were performed, depending on whether a full or partial view was used, and if the resolution of the scan and the database object models was the same or not:

1. Full View Same Resolution Experiment (FVSR).
2. Full View Different Resolution Experiment (FVDR).
3. Partial View Same Resolution Experiment (PVSR).
4. Partial View Different Resolution Experiment (PVDR).

**Figure 8:**
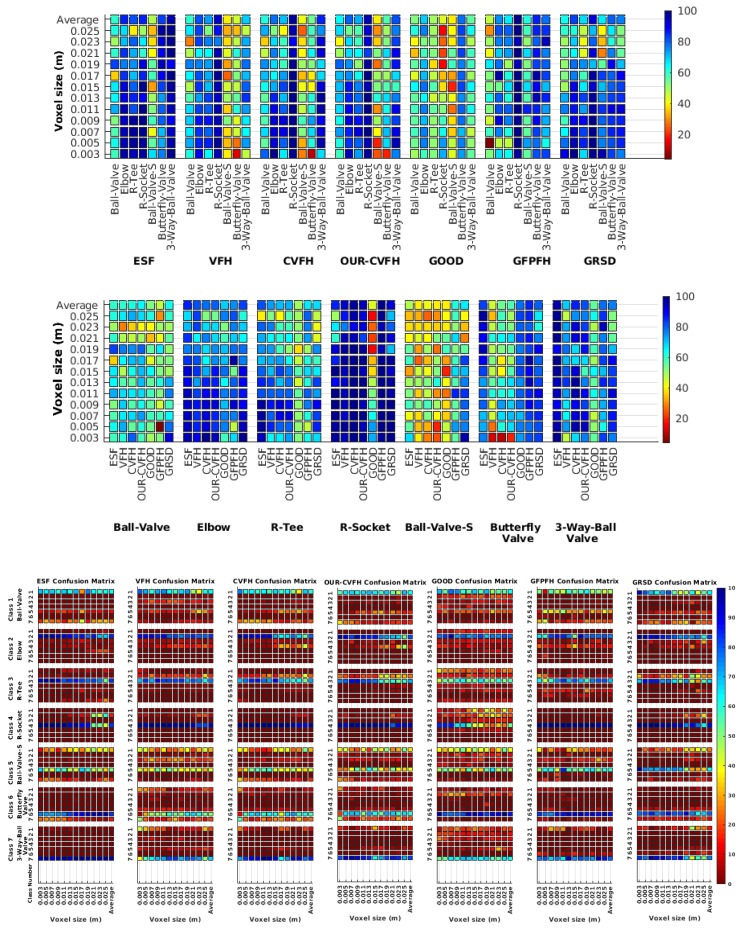
Average of recognition per resolution for all descriptors, using full views and having the same resolution for the model and the measurement: (**Top**) Grouped by descriptor; (**Middle**) Grouped by object; (**Bottom**) Confusion Matrix.

**Figure 9:**
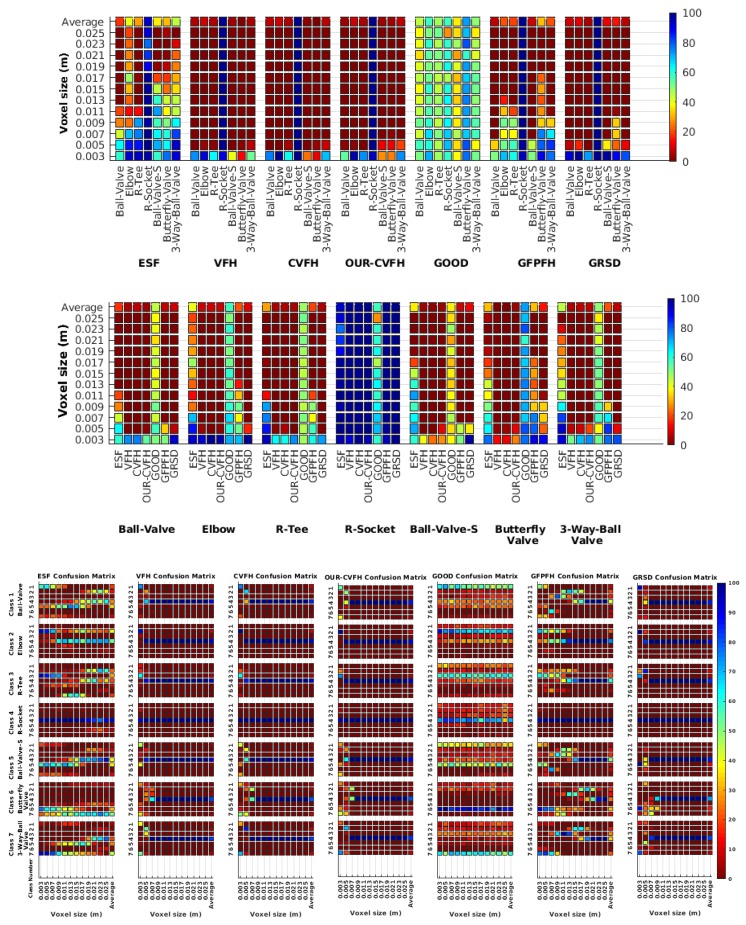
Average of recognition per resolution for all descriptors, using full views and having the different resolution for the model and the measurement: (**Top**) Grouped by descriptor; (**Middle**) Grouped by object; (**Bottom**) Confusion Matrix.

**Figure 10:**
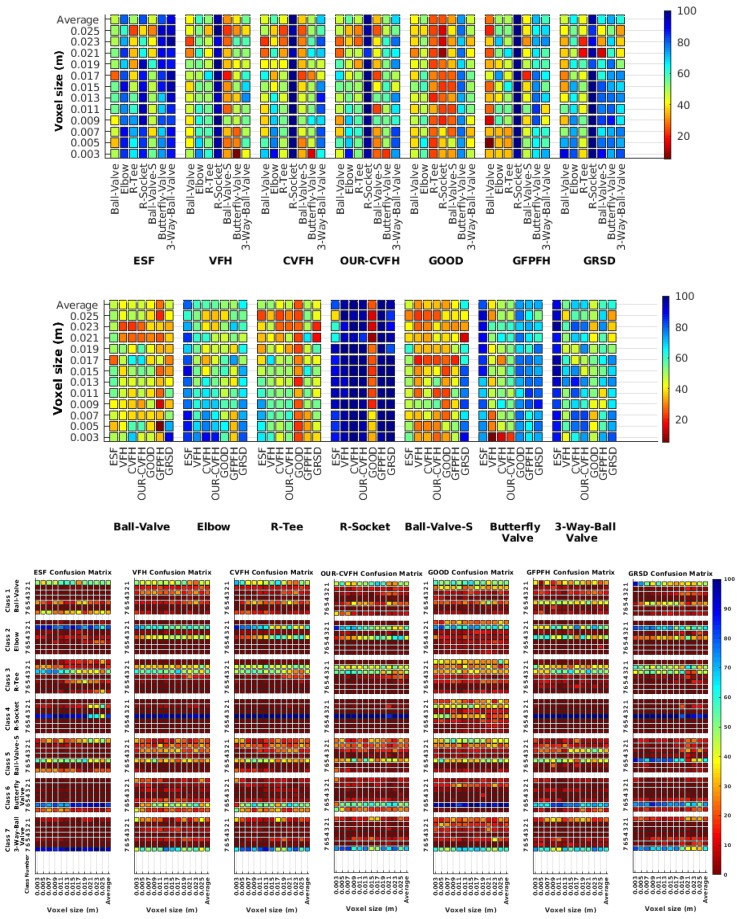
Average of recognition per resolution for all descriptors, using partial views and having the same resolution for the model and the measurement: (**Top**) Grouped by descriptor; (**Middle**) Grouped by object; (**Bottom**) Confusion Matrix.

**Figure 11:**
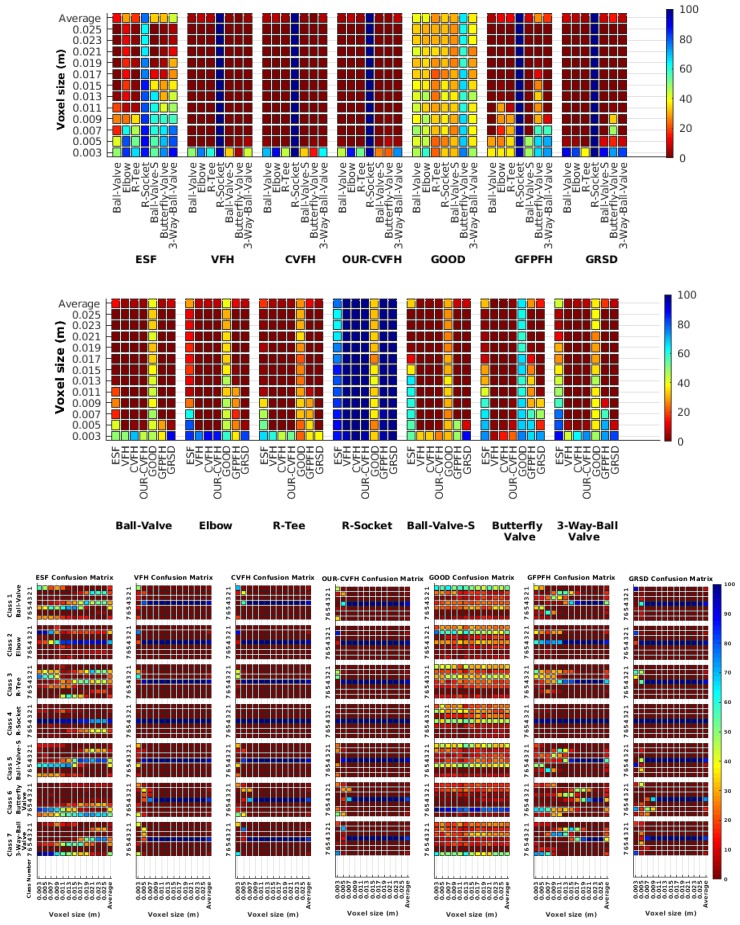
Average of recognition per resolution for all descriptors, using partial views and having different resolution for the model and the measurement: (**Top**) Grouped by descriptor; (**Middle**) Grouped by object; (**Bottom**) Confusion Matrix.

Each experiment involved 100 Montecarlo runs, and their results are respectively shown in Figure 8, Figure 9, Figure 10 and Figure 11. Moreover, the average results among all the objects for all the resolutions are shown in Table 4. Finally, a summary of the results taking into account all the objects and all the studied resolutions is shown in Table 5.

### 6.1. Difference of Resolution Between the Scan and the Object Model in the Database

Figure 8 shows the average recognition for every descriptor and every resolution when both the database and the measured scan contain full views of the object and have the same resolution (FVSR). On the other hand, Figure 9 shows the same data for a second set of experiments (FVDR), when the resolution used for the object model in the database and the resolution of the measured scan are different. The blue color (indicating a high average recognition rate) the top row Figure 8 is significantly more present than in Figure 9, meaning that better results are achieved using the same resolution instead of different ones. The same can be appreciated in Figure 10 and Figure 11 for the more realistic case when the measured scan shows only a partial view of the object (PVSR and PVSR experiments). This can be clearly observed in Figure 4 which shows the average recognition rate for all the descriptors and all the resolutions for full and partial object views in both cases, with the same and different resolutions. The lower row of the figure clearly shows a significant drop in the average recognition rate for resolutions beyond 0.005 for both cases, partial and full object views. Finally, the last column of Table 5 (*Average over descriptors*) shows the recognition rate averaged for all objects, all resolutions and all the descriptors. When the same resolution is used the recognition rate is 62.9 reducing to only 26.6 when the resolutions differ.

### 6.2. Scan Resolution

To study how the resolution affects the recognition let us have a look at the upper row of Table 4. In both cases, full view (shown at left side) and partial view (shown at right side), it can be appreciated how the performance decreases with the reduction of the resolution for the two better descriptors: ESF and GRSD. Interestingly, the performance of the GOOD descriptor remains almost constant across the different resolutions, even when different resolutions are used among the database object models and the scan. The behaviour for the rest of descriptors is more arbitrary not showing a clear trend. The lower row of Table 4 clearly shows how the performance decreases as the difference between the database model and the measured scan increases(the object model is at resolution 0.003 and the input scan resolution is varied during the experiment), with the remarkable exception of the GOOD descriptor.

### 6.3. Full vs. Partial Object View

The importance of measuring a view as wide as possible is shown in Table 4 as well. The left-hand column of the table corresponds to the case when the full view of the object is observed (FVSR and FVDR). The right-hand column corresponds to a partial view (PVSR and PVDR). In both cases, for same and different resolution between the model and the measured scans, a ≈10% decrease in the average recognition rate is observed. This decrease in the average recognition is confirmed in Table 5 where the averaged recognition rate for all descriptors is 8.4% better for full view (48.9) than for partial view (40.5%). Unfortunately, the observation of partial views is the more realistic case so its results should be considered more representative of the reality.

### 6.4. Best Descriptor

Qualitatively, the best performing descriptor can be inferred from the top row of Figure 8, Figure 9, Figure 10 and Figure 11. The descriptors whose object×voxel_size grid is predominantly blue are the ones performing better, while those predominantly yellow, orange or red are progressively the worst ones. In the results of the FVSR experiment, Figure 8, it can be clearly appreciated that GRSD and ESF are the best descriptors, while GOOD is the worst one. When partial views are used instead, PVSR experiment, a decrease of performance (colors shifted towards green) can be seen, but with essentially the same results. ESF and GRSD continue being the best descriptors while GOOD is the one with lower performance. In the FVDR and PVDR experiments, when different resolutions between the model and the scan are used the scenario is totally different. In this case, only the GOOD descriptor is able to provide significant results.

The results may also be analyzed quantitatively. Table 5 shows how well each descriptor performed (averaged among objects and resolutions). The results averaged among all the experiments using full views, show clearly that the best performing descriptor is ESF (59.6%) followed by GOOD (53.5%) while CVFH (43.9%) is the worst one. If partial views are used instead, the best descriptor is still ESF (50.9%), followed in this case by GRSD (42.4%), with VFH (35.0%) being the worst one. If we focus only on the dimension related to the same/different resolution, then, using the same resolution ESF (72.5%) is the best one followed by GRSD (69.4%) with GOOD (48.5%) being the one performing worst. When different resolutions are used instead, GOOD (45.1%) becomes the best one followed by ESF (38.0%), and VFH (17.6%) the worst. If we average the results among all the experiments (last row of the table), we conclude that ESF (55.3%) is the one performing better in general followed by GOOD (46.8%), and VFH (38.4%) the worst performing one. In our opinion, the most relevant results corresponds to the PVSR experiment because having full views is not always possible and, at least in our case, having the same resolution is always easy. In this case, ESF (67.6%) and GRSD (63.8%) are the best descriptors and GOOD (41.8%) is the one providing poorest results.

### 6.5. Object Confusion

Besides looking at the average recognition rate and, in order to understand the descriptor capabilities for object recognition, it is good to examine the confusion matrices. For every object, they show the object-class that is recognized, but also, when it is mis-recognized, which are the classes that generate the confusion. It is worth noting, hence, that the smaller the recognition rate the higher the confusion. Figure 8, Figure 9, Figure 10 and Figure 11 show, in their bottom row, the confusion matrices for the different experiments. To extract conclusions about confusion, regardless of the descriptor, we averaged the results among all the descriptors in Table 6. Examining them we can extract the following general conclusions:

1. The lower the resolution the higher the confusion. This can be appreciated in Table 4 since the recognition rate decreases with the resolution.
2. The recognition rate is higher than the mis-recognition (The addition of all the confusion percentages) only when the same resolution is used. Using the same resolution leads to less confusion (60.3% average recognition rate), while using different resolutions leads to significantly higher confusion (27% average recognition rate).
3. The use of full views also leads to less confusion (49.4 average recognition rate) than using partial views (37.9 average recognition rate).

**Figure 12:**
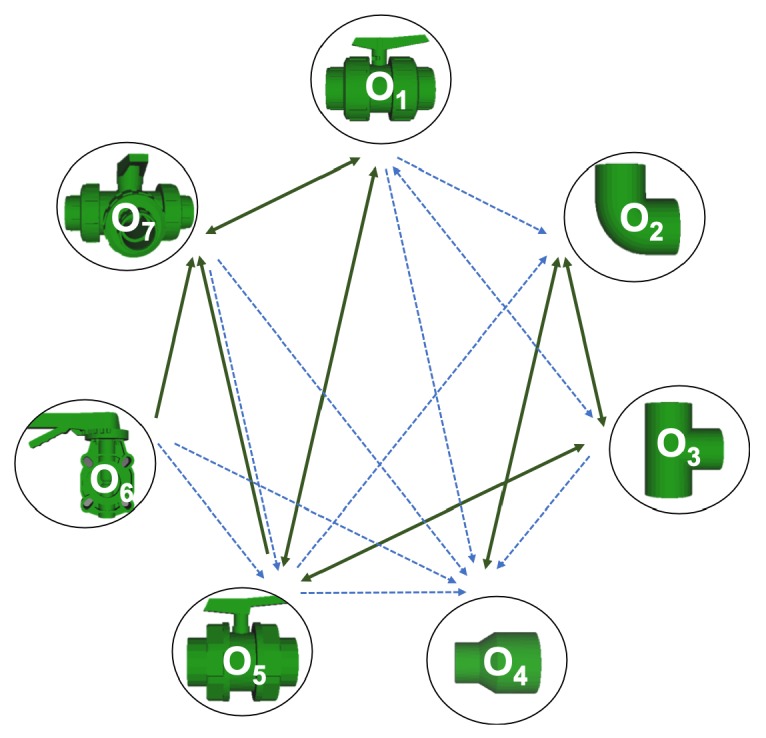
Confusion per Object Graph.

Now, several relevant questions arise:

1. *Which is the descriptor provoking most confusion?* Let us focus on the results when using the same resolution which we consider the most interesting ones. In this case, see Table 5, the descriptor with the lowest recognition rate is GOOD, being hence the one leading to higher confusion. This can be confirmed looking at its confusion matrices in Figure 8 and Figure 10. On the other side of the spectrum we find ESF and GRSD which have good recognition rates (72.5% and 69.4% respectively), leading to less confusion as can be appreciated in Figure 8 and Figure 10.
2. *When confusion arises, which are the objects more prone to be confused?* As stated above, the two most interesting scenarios are the ones corresponding to the same resolution, FVSR and PVSR. Figure 12 shows how objects are confused in those scenarios. The green arrows correspond to the confusions appearing (those whose percentages is higher than 5%) when using full views. In this case, most of the confusion appears either among the valves ($O_{1},\;O_{5},\;O_{6},\;O_{7}$) or among the Elbow, R-Tee and the R-Socket objects. Moreover The R-Tee is also confused with Ball-Valve-S ($O_{5}$). When partial views are used instead, the blue arrows add on top of the green ones showing new confusions (The black ones still exist with partial views), making the object identification more challenging. The graph shows clearly how the use of partial views leads to more confusion.

### 6.6. Gaussian Noise

In this section Gaussian noise is introduced in the simulation to study how it affects the recognition rate. Only the case of the same resolution is evaluated since from previous results it is clear that it provides the best results. The two experiments, the full and partial views, were considered. In both cases 100 Montecarlo runs were executed over 12 different resolutions for 6 different noise levels (see Table 3). Moreover, the average results among all the objects for all the resolutions are shown in Table 7. Finally, a summary of the results taking into account all the objects and all the studied resolutions is shown in Table 8.

**Figure 13:**
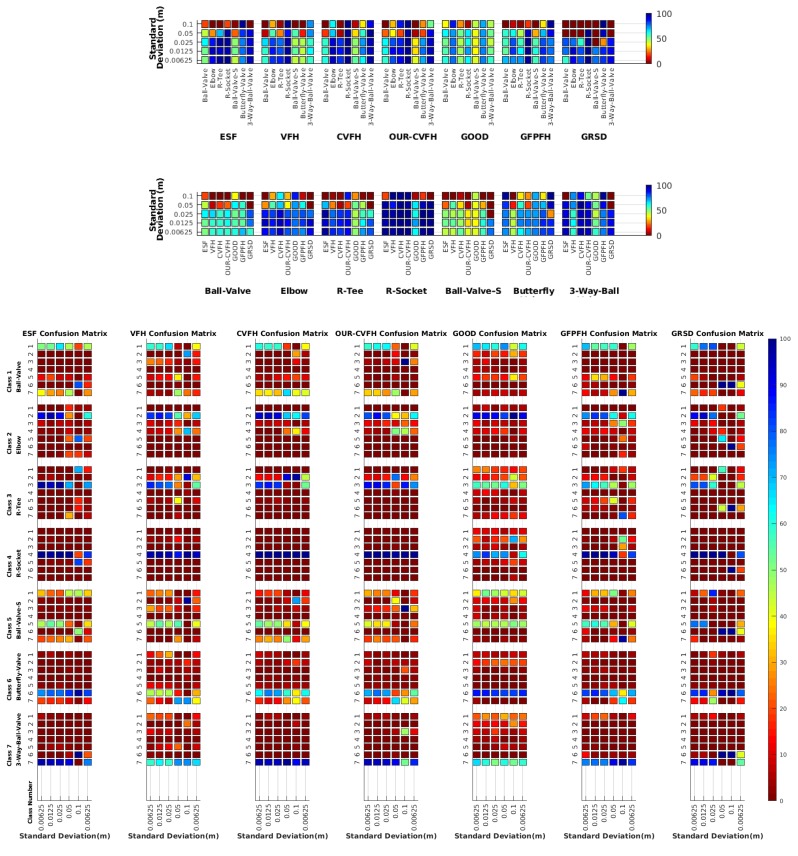
Average of recognition for the resolution 0.007 for all descriptors, using full views and having the same resolution for the model and the measurement: (**Top**) Grouped by descriptor; (**Middle**) Grouped by object; (**Bottom**) Confusion Matrix.

**Figure 14:**
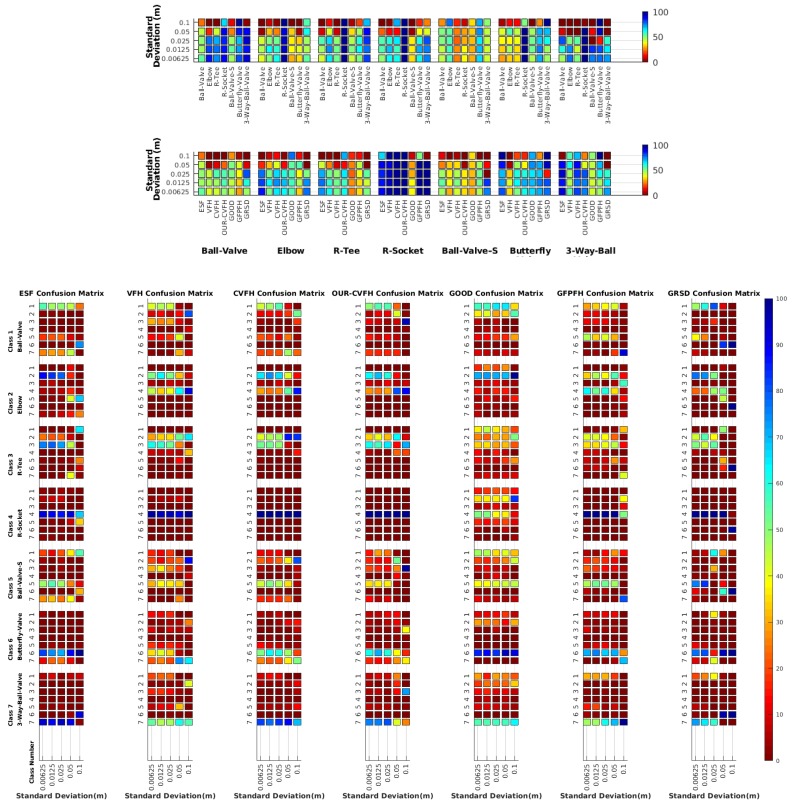
Average of recognition for the resolution 0.007 for all descriptors, using partial views and having different resolution for the model and the measurement: (**Top**) Grouped by descriptor; (**Middle**) Grouped by object; (**Bottom**) Confusion Matrix.

Assuming 0.007 is the resolution for the scanner used in the real experiments reported in the next section, Figure 13 and Figure 14 show the recognition rate, for all the descriptors, and every noise level, at this resolution, respectively for:

1. Noisy Full View Same Resolution Experiment (NFVSR).
2. Noisy Partial View Same Resolution Experiment (NPVSR).

#### 6.6.1. Scan Resolution

Table 7, shows the recognition rate averaged for all objects, detailed for every resolution. It is interesting to note that while most of the descriptors’ performance decreases with the resolution as well as with the noise, some of them show a very poor result for the high-resolution and high-noise combination. This is the case for the GRSD and ESF (at higher noise ratios). We attribute this to the fact that ESF is based on the shape function computing distances and angles between random points and GRSD used the radial relationships to describe the geometry of points at each voxel. Accordingly, the impact of these two factors could be amplified when both resolution and noise level are high.

#### 6.6.2. Full vs. Partial Object View

As observed in the previous experiments, better recognition rates are achieved using full views (see Table 8). The improvement with respect to the use of partial view ranges from ≈15% at σ∈[0–0.0125], ≈15% at σ∈[0.025–0.05], and ≈3% at σ=0.1.

#### 6.6.3. Best Descriptor

The results reported in Table 8 show that ESF is either the best, or the second best, descriptor except for the highest noise level where its performance drops significantly, making it one of the 2 worst performing descriptors. GRSD works well at low noise levels but its performance drops significantly when the noise is medium to high. GOOD is the worst performing one at low noise levels, but performs well at high noise. VFH performs poorly across the whole noise spectrum while CVFH, OUR-CVFH and GFPFH present intermediate performance levels. On the other hand, Table 9 shows the recognition rate, for all the descriptors, averaged by object, for the resolution 0.007. This is the assumed resolution for the scanner used in the real experiments reported in the next section. There we can see that for full view and low noise (σ=0.00625), which is the case corresponding to our sensor, GRSD is the best descriptor followed by ESF, GFPFH, OUR-CVFH and CVFH. GOOD and VFH are the worst ones. If we go to the other extreme, high noise (σ=0.1), GOOD becomes the best descriptor with results close to CVFH and OUR-CVFH. GRSD is the worst performing one closely followed by ESF, GFPFH and VFH. For partial views and low noise ESF and GRSD are the best ones, followed by OUR-CVFH and CVFH (medium-high performance), VFH and GFPFH (medium-low performance) and GOOD (worst performance). At high noise levels the best one is GOOD followed by GFPFH, CVFH and ESF with less performance while VFH and GRSD are the worst ones.

#### 6.6.4. Object Confusion

**Figure 15:**
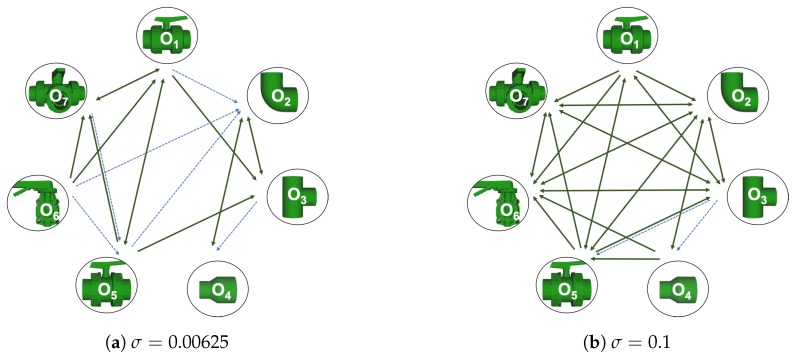
Object Confusion Graph.

Table 10 shows the confusion tables for all object and all noise combinations averaged for all the descriptors. Notice that the cells marked in blue are those above 5%. The *‘average recognition for all objects’* column shows that on average, for all the objects, the recognition works well for noise levels equal to or below 0.025. Beyond that, the recognition rate falls below 50%. It can be observed, that for σ≤0.025 all the objects are recognized with a recognition rate over 50% for both cases, full and partial views, except for the Ball-Valve and the Ball-Valve-S objects. For them, the recognition rate is sometimes below 50%, especially for partial views. It can also be appreciated, that the number of cells with a percentage of confusion over 5% (cells in blue) increases when using partial views than when using full views. This effect is observed in almost every object indicating that the use of partial views leads to more confusion. For σ≥0.05 the recognition rate decrease significantly below 50% with the exception of the R-Socket and the Butterfly-Valve objects. Figure 15 shows the confusion graph for the lowest and highest noise cases. For the first case, it can be seen how new confusion links appear when partial views are used instead of full ones. This is consistent with the results of the previous section. In the case of high noise, most of the confusion actually appears even using full views, and only two more confusions appear when using partial views. As expected, this shows how increasing the noise also increases the percentage of confusions.

## 7. Results on Underwater Testing

**Figure 16:**
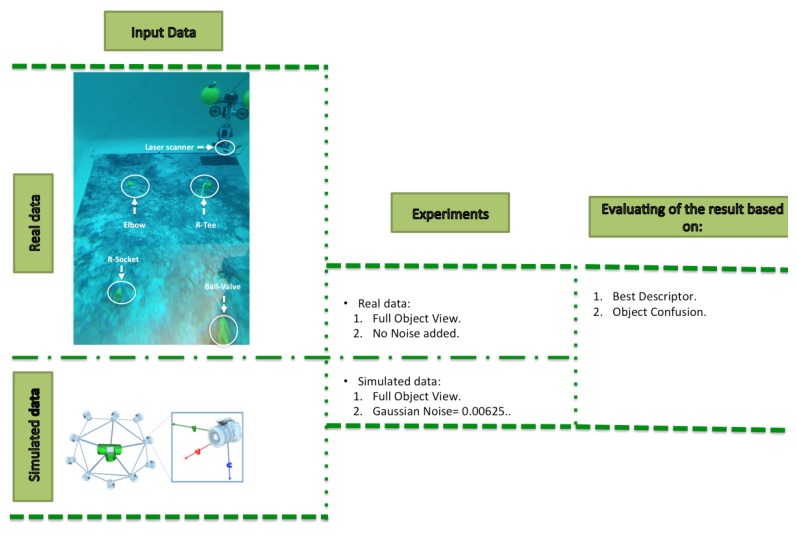
Diagram of the real versus simulated experiment. The upper left figure illustrates the experimental setup, with the Girona 500 AUV deploying an underwater laser scanner inside a water tank, where the four objects were laid on the bottom.

In this section we present experimental results and compare them with the previous simulations. The setting and the analytic process of the experiment are summarized in Figure 16. We took advantage of an already collected dataset which was previously used for semantic Simultaneous Localization And Mapping (SLAM) [73]. The data was collected using an in-house-developed laser scanner [28] mounted on GIRONA500 Autonomous Underwater Vehicle (AUV) [74], which was performing a trajectory in an small water tank. The experiment involved 25, 29, 48 and 48 observations of full view scans corresponding to the Ball-Valve, Elbow, R-Tee, and R-Socket objects respectively. Although the data-set only used 4 out of the 7 objects used in this survey, we think the results are representative.

### 7.1. Real vs. Simulated Rresults

To compare the results obtained in the real experiment to the simulation results, a supplementary simulation was performed. The experiment involved 100 Montecarlo runs, as in the previous simulations, considering solely the four objects that were involved in the real experiment namely: Ball-Valve, Elbow, R-Tee, and R-Socket. The simulation parameters *<objects = 4, resolution = 0.007, noise = 0.00625, view = Full>* represent the case closest to the real data. The simulated scans were generated assuming a distance to the object d=1.11±0.56 m, and yaw&roll angles were varied between −0.4 and 7.4 degrees, while the pitch ranged between −2.2 and 3.9 degrees. The simulated values were chosen randomly within those ranges, corresponding to the ones observed in the real experiment.

Table 11, shows the corresponding percentages of how many times each different object class was recognized for both real and simulated runs, respectively, so they can be compared. Notice that the yellow cells represent the objects with their respective class number.

As expected, the recognition works better with the simulated scans than with the real ones. This is understandable, taking into account that real scans can be potentially affected by errors due to: (1) non-perfect scanner calibrations and (2) motion induced distortion. The latter may be significant, since the scanner works by steering a laser beam (which takes time to scan the scene) and assumes the sensor is static during the process (which is never the case since the robot is floating). To illustrate the problem we can look at Table 12. The top row shows a successful recognition example corresponding to the ESF descriptor. At the left, is shown a laser scan of a Ball-Valve (in black) and the corresponding object view (in red) matched in the data base. At the right, both histograms, the one corresponding to the scan and the one corresponding to the matched view show good agreement. On the other hand, the bottom row shows an example of mis-recognition of an R-Tee object using the GFPFH descriptor. At the left, is shown the laser scan (in black), the matched object view in the data base (in red), and the most similar view in the database manually selected by us (in blue). The corresponding histograms are shown at the right side of the bottom row. Although we perceive the black scan to be closer to the blue view than to the red one, the difference is evaluated quantitatively by the corresponding histograms, and it is clear that the black histogram is closer to the red one than to the blue. It is worth noting the distortion present in the black scan, which is probably the origin of the mis-recognition.

#### 7.1.1. Best Descriptor

Table 11 shows the average recognition rate for the real and the synthetic experiments, highlighting in green the two better performing descriptors and in red the two worst ones. In the real experiments, the best performing descriptor is CVFH (95.4%) followed by OUR-CVFH (91.5%). This result is in agreement with the simulated one (both at 100%). The worst one is GFPFH (54.1%) followed by GOOD (58.0%) in the real experiment, with GOOD being (75.0%) and VFH (88.0%) in the simulated ones. We think that this disagreement is due to the fact that our results for GFPFH differ significantly between reality (54.1%) and simulation (96.8%), probably affected by problems like the one commented above which is illustrated in Table 12.

#### 7.1.2. Object Confusion

**Figure 17:**
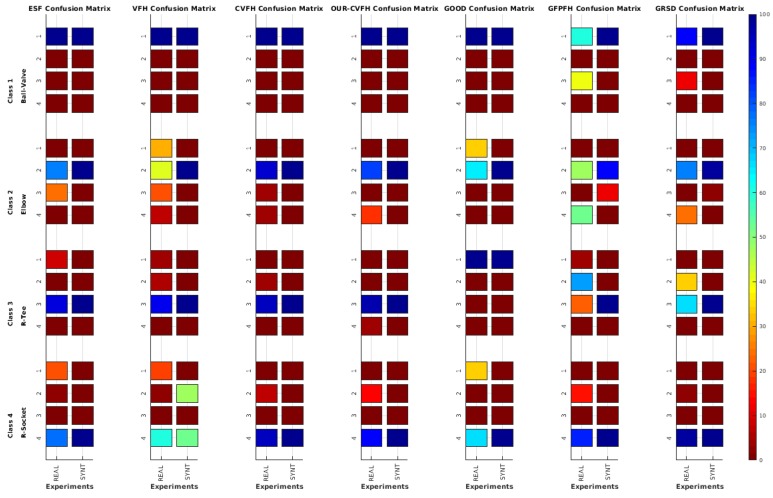
Confusion Matrix for the real and synthetic data, for all descriptors, where the first column corresponds to real data and the second to synthetic data.

Figure 17 and Table 11 show the confusion matrices for the real and the synthetic experiments. First it is worth noting that, as expected, real experiments lead to more confusion than synthetic ones. Second, focusing on the real results, it can be observed that in general (averaging over all descriptors), the Ball-Valve is the most easily recognizable object, while the R-Tee and the elbow were the objects leading to more confusion (average recognition of 65.9% and 69% respectively). Nevertheless, focusing on CVFH and OUR-CVFH (the best performing descriptors), we can see that the first one does an excellent job having only one confusion beyond the 5% boundary (R-Socket confused with Elbow), while the second one adds a second confusion beyond the 5% limit (Elbow confused with socket). Finally, it called our attention to the experimental and simulated results corresponding to the GOOD descriptor and the R-Tee object. In Table 11, it can be appreciated how it fails to recognize the object (0%) and confuses it (100%) systematically with the Ball-Valve. We attribute this result to the fact that GOOD works based on the orthographic projection on XoZ, XoY, and YoZ. Checking the object-database (Figure 2) it can be observed that there is no R-Tee view corresponding to the top view observed by the laser in the real experiment, while there are two views of the Ball-Valve in the database which projected onto XoZ, XoY, and YoZ look similar to the top view of the R-Tee Object. This problem illustrates how important it is to have representative views in the database of those objects which will be observed. Please note that this problem did not arise in the FVSR and PVSR experiments, since in those cases, the scans were not forced to be taken from the top as happens in the water-tank experiment where a downward-looking laser scanner was used.

This could explain the divergence of the simulated and real results reported for some of the entries of Table 11.

## 8. Interpretation of Results

This section provides further interpretation of the results of all the experiments performed in this study.

### 8.1. Simulated Data

**Figure 18:**
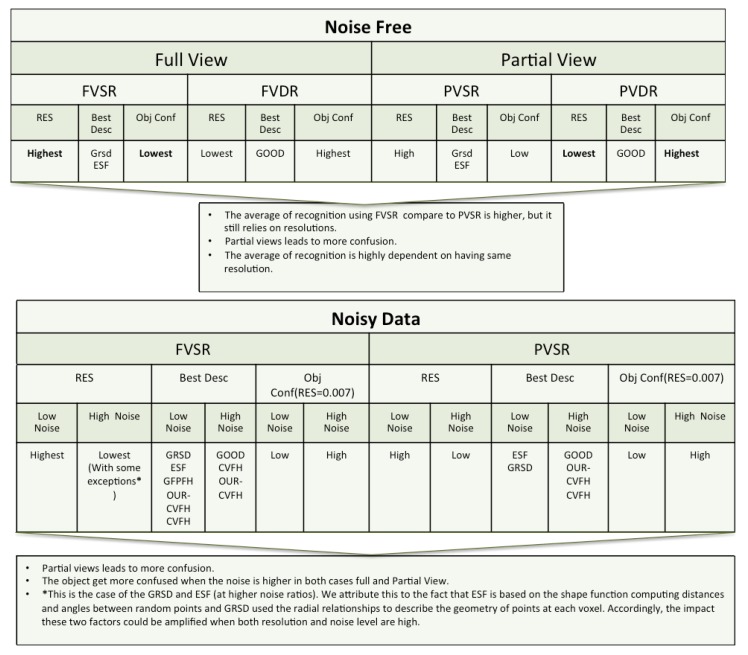
Summary of the result section for the simulated data. Res, Best Desc and Obj Conf represent respectively: Resolution, Best Descriptor and Object Confusion

The results detailed in Section 6, obtained using simulated data, are summarized in Figure 18.

The following points contain the main findings from the experiments, under testing conditions involving the use of: full or partial views, same or different resolution of the scan and the database object models, and full or partial views under added noise.

1. Full View Same Resolution and Partial View Same Resolution Experiment
  The best performance in both cases was achieved using ESF and GRSD. The average of recognition is slightly better using the full view than the partial one, where the trend of the recognition accuracy with respect to the descriptors was monotonic in both cases.
2. Full View Different Resolution and Partial View different Resolution Experiment
  Only the GOOD descriptor provided significantly valid results. The change of resolution did not affect the performance of the descriptor in either of the scenarios (FVSR and PVSR). This invariance of the performance regarding the resolution was reported in the original work of the authors in [68].
3. Same resolution versus different Resolution Experiment
  From the cases of FVDR and PVDR, changing the resolution in the database and test led to poor recognition. Conversely, the cases of FVSR and PVSR, show that having the same resolution in both the database and test leads to higher recognition rates.
4. Full Views versus Partial Views
  Predictably, using full object views instead of just a partial view, leads to better results. This behaviour is somehow expectable, given the global nature of the methods tested, and the fact that the descriptors in the database were always computed from full views. Additionally, from the confusion matrices, the objects that are prone to confusion when using partial views are a superset of the ones for the case of using full views.
5. Noisy Full View versus Noisy Partial View
  In these experiments only the case where the resolution of the database and test are similar were taking into account. As general assessment, the results of NFVSR versus NPVSR follow the same trend as discussed in the noise-free experiment, where the performance of the descriptor decreases with lower resolution and higher noise ratio, except for GRSD and ESF where the performance of the descriptors decreased for high resolution and noise. We attribute this difference to the fact that ESF is based on the shape function computing distances and angles between random points, while GRSD used the radial relationships to describe the geometry of points at each voxel. Accordingly, the impact of these two factors could be amplified when both resolution and noise level are high.
  As a specific assessment, the confusion matrix for the resolution=0.007, which is the resolution of the laser scan used in the real experiment, was computed at a different noise level. The results showed that the object got more confusion at a high noise level compared to a low level.

### 8.2. Underwater Data

**Figure 19:**
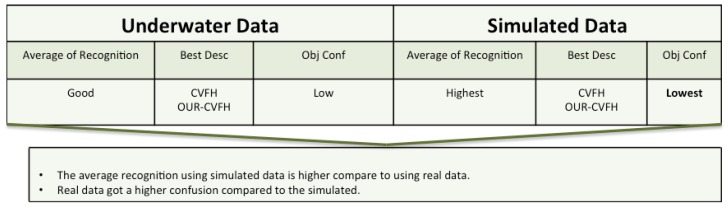
Sumnary of the results section for real versus simulated data.

The figure below (Figure 19) summarizes the results based on real underwater versus simulated data.

From the results presented in Section 7, it is worth noting that CVFH and OUR-CVFH were the two best performing descriptors in both real and simulated data. Also that the recognition based on the descriptors GFPGH and GOOD gave slightly different values when using the real and the simulated data. These differences can be explained by several factors:

- Real data inevitably suffers from noise generated from the changes of the position of the laser during the acquisition of the point cloud, causing a distortion of the object shape and leading to a different descriptor representation. These motion distortions were present in real but not in simulated data.
- Most of the object descriptors used in this study are based on use of a surface normals. Noise causes a modification in the surface which causes a change in the normal for each point.

## 9. Conclusions

This paper presented a survey and comparison of global descriptors for 3D object recognition purposes when a 3D model of the object is available a priori. Because our focus of interest is centered in underwater IMR applications, we selected seven representative objects commonly present in submerged pipe infrastructures. Using their CAD models, we set up a database containing 12 views of each object. Next, seven global descriptors available in the Point Cloud Library were selected and compared exhaustively in simulations and through water tank experiments. Different criteria were evaluated: (1) the use of partial vs. global views, (2) the use of same vs. different resolution between the object model and the input scan, (3) the effect of resolution and (4) the effect of noise.

Our results demonstrate that, as intuition suggests, using global views provides better results than using partial views. Less intuitive is the conclusion that using the same resolution in the views of the database and the input scan leads to significantly better results. The combination of both cases is therefore the best scenario: full view/same resolution. When the resolution of the scan is analyzed, in general, for most descriptors the higher the resolution the better the recognition rates. Hence decreasing the resolution leads to a decrease in the performance, with the exception of the GOOD descriptor whose performance remains constant over the studied resolutions.

Another parameter studied was the noise. In this case, the results follow intuition and the higher the noise the worse the recognition rate and the higher the object confusion. The exception is again the GOOD descriptor which is the best one for high levels of noise.

It is not straightforward to single out the best performing descriptor, since this depends on the particular combination of the different parameters studied. Therefore, the numerous graphs provided for each one of them may help other researchers to make their own decisions based on the particular constraints of their own application.

## 10. Future Work

**Figure 20:**
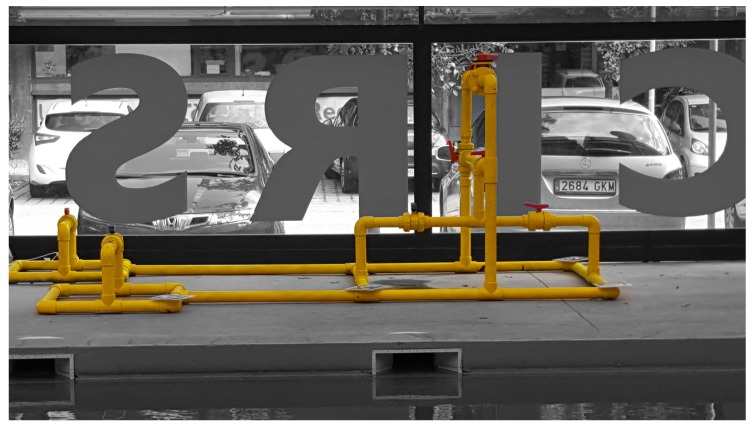
Structure of PVC objects.

A central goal of our work is the use of a real-time laser scanner mounted on an intervention AUV to detect, identify and locate objects in the robot’s surroundings, and to use this information to allow the robot to decide which manipulation actions may be performed on each type of object. Therefore our next step is going to focus on implementing a method to recognize objects within a point cloud which may contain several of them. This will require a method to segment the different objects so that they can be recognized later on. Once recognized they will then be located and introduced into a SLAM algorithm to set-up a semantic map of the robot environment. As an example, Figure 20 illustrates a test structure containing multiple object instances that is currently being used for this purpose.

## Figures and Tables

**Table 1:** Summarized characteristics of the seven descriptors used in this paper. The “based on” column indicates if the descriptor evolved directly from another approach. The “use of normals” indicates whether the method uses surface normals for computing the descriptor, while the last column indicates the length of the descriptor vector.

Descriptor	Main Characteristics		
	Based on	Use of Normals	Descriptor Size
Global Orthographic Object Descriptor (GOOD)-2016—[68]	-	No	75
The Ensemble of shape functions (ESF)-2011—[64]	Shape function [69]	No	640
Global Radius-based Surface Descriptors (GRSD)-2010—[54]	RSD [55]	Yes	21
Viewpoint Feature Histogram (VFH)-2010—[53]	Fast Point Feature Histogram (FPFH) [46]	Yes	308
Global Fast Point Feature Histogram (GFPFH)-2009—[33]	Fast Point Feature Histogram (FPFH) [46]	Yes	16
Clustered Viewpoint Feature Histogram (CVFH)-2011—[34]	VFH [53]	Yes	308
Oriented, Unique and Repeatable CVFH (OUR-CVFH)-2012—[62]	CVFH [34]	Yes	308

**Table 2:** Polyvinylchloride (PVC) pressure pipes objects used in the experiments.

PVC Objects	Id Name	Size (mm^3^)	PVC Objects Views (12)
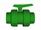	1-Ball-Valve	198×160×120	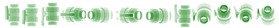
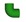	2- Elbow	122.5×122.5×77	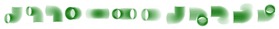
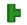	3- R-Tee	122.5×168×77	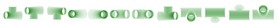
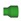	4- R-Socket	88×75×75	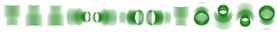
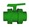	5- Ball-Valve-S	174×160×118	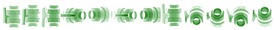
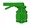	6- Butterfly-Valve	287.5×243×121	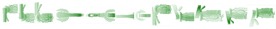
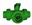	7- 3-Way-Ball-Valve	240×160×172	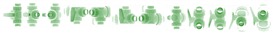

**Table 3:** Views of the Ball-Valve scan with different resolution and noise levels used in the experiment: the line “VX” indicates the different voxel size resolutions and column “σ” indicates the different standard deviations of the added noise. All values are in meters.

	VX (m)	0.003	0.005	0.007	0.009	0.011	0.0013	0.015	0.017	0.019	0.021	0.023	0.025
σ (m)													
0		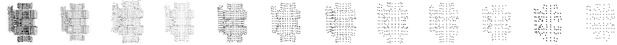											
0.00625		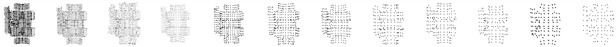											
0.0125		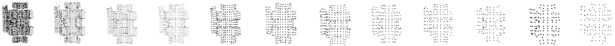											
0.025		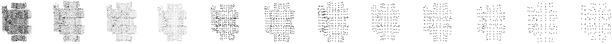											
0.05		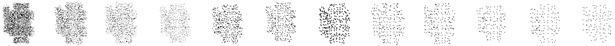											
0.1		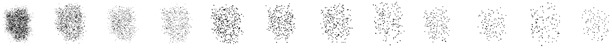											

**Table 4:** Average of recognition per resolution for all descriptors: (top-left) Using full object views and the same resolution between the model and the measurement; (top-right) Using partial object views and the same resolution between the model and the measurement; (bottom-left) Using full object views and different resolution between the model and the measurement; (bottom-right) Using partial object views and different resolution between the model and the measurement.

	Full Object View	Partial Object View
**Same Resolution**	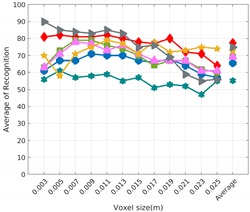	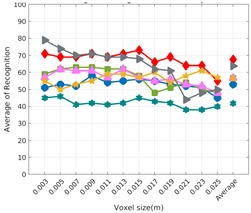
**Different Resolution**	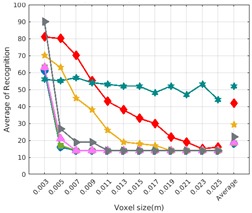	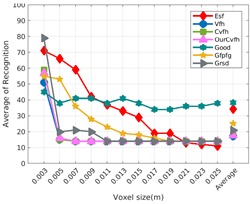

**Table 5:** Summary of results for all the objects and all the resolutions. The best descriptor is marked in green, while the worst one is marked in red.

Experiment	View	Resolution	Descriptors							Average Over Descriptors
			ESF	VFH	CVFH	OURCVFH	GOOD	GFPFH	GRSD	
FVSR	Full	Same	77.4	65.3	69.5	69.3	55.1	72.8	75.0	69.2
FVDR		Different	41.8	18.1	18.3	18.7	51.9	29.3	22.3	28.6
PVSR	Partial	Same	67.6	52.8	56.7	56.8	41.9	56.5	63.8	56.6
PVDR		Different	34.3	17.2	17.8	17.8	38.3	25.3	21.0	24.5
FVSR/FVDR	**Average Over**	Full View	59.6	41.7	43.9	44.0	53.5	51.0	48.6	48.9
PVSR/PVDR		Partial View	50.9	35.0	37.3	37.3	40.1	40.9	42.4	40.5
FVSR/PVSR		Same Res	72.5	59.1	63.1	63.0	48.5	64.6	69.4	62.9
FVDR/PVDR		Diff Res	38.0	17.6	18.1	18.2	45.1	27.3	21.6	26.6
**FVSR/FVDR/** **PVSR/PVDR**	**Full Average**		55.3	38.4	40.6	40.6	46.8	46.0	45.5	44.7

**Table 6:** Summary of confusion matrices for all the objects and all descriptors averaged along resolutions. Marked in blue are those confusions greater than 5% which where used to build the object confusion graph of Figure 12.

**Experiment**	**View**	**Resolution**	**Objects**																											
			**Ball Valve**							**Elbow**							**R-Tee**							**R-Socket**						
			**1**	2	3	4	5	6	7	1	**2**	3	4	5	6	7	1	2	**3**	4	5	6	7	1	2	3	**4**	5	6	7
FVSR	Full	Same	**58,8**	2,8	4,3	0,7	16,7	0,5	16,3	1,6	**78,8**	6,8	10,7	1,4	0,6	0,1	4,6	15,6	**69,6**	1,6	5,3	0,9	2,3	2,8	8,3	1,3	**86,3**	1,0	0,1	0,2
FVDR		Different	**13,5**	7,5	3,8	62,3	8,0	1,1	3,8	1,7	**22,0**	1,5	73,3	0,8	0,6	0,1	3,1	8,6	**17,0**	64,3	2,3	1,0	3,8	1,5	2,1	1,7	**92,7**	1,0	0,8	0,2
PVSR	Partial	Same	**36,6**	9,9	8,7	13,5	19,8	1,1	10,4	3,0	**51,5**	3,2	39,0	2,0	0,7	0,7	5,6	28,5	**38,6**	18,6	5,7	0,7	2,2	2,7	7,8	1,2	**85,4**	1,6	0,7	0,6
PVDR		Different	**11,3**	7,9	3,3	63,7	8,5	1,5	3,7	2,9	**15,5**	1,4	77,2	1,4	0,9	0,8	3,9	11,1	**10,6**	66,8	3,3	1,1	3,2	2,6	4,1	1,9	**87,0**	1,2	2,5	0,6
FVSR/ FVDR	**Average Over**	Full View	**36,1**	5,2	4,0	31,5	12,4	0,8	10,0	1,6	**50,4**	4,1	42,0	1,1	0,6	0,1	3,9	12,1	**43,3**	32,9	3,8	1,0	3,1	2,1	5,2	1,5	**89,5**	1,0	0,4	0,2
PVSR/ PVDR		Partial View	**24,0**	8,9	6,0	38,6	14,2	1,3	7,1	2,9	**33,5**	2,3	58,1	1,7	0,8	0,7	4,7	19,8	**24,6**	42,7	4,5	0,9	2,7	2,7	6,0	1,6	**86,2**	1,4	1,6	0,6
FVSR/ PVSR		Same Res	**47,7**	6,3	6,5	7,1	18,3	0,8	13,3	2,3	**65,2**	5,0	24,8	1,7	0,7	0,4	5,1	22,1	**54,1**	10,1	5,5	0,8	2,3	2,8	8,1	1,3	**85,9**	1,3	0,4	0,4
FVDR/ PVDR		Diff Res	**12,4**	7,7	3,5	63,0	8,3	1,3	3,8	2,3	**18,8**	1,4	75,2	1,1	0,7	0,5	3,5	9,9	**13,8**	65,5	2,8	1,1	3,5	2,0	3,1	1,8	**89,9**	1,1	1,7	0,4
**FVSR/** **FVDR/** **PVSR/** **PVDR**	**Full Average**		**30,1**	7,0	5,0	35,0	13,3	1,1	8,5	2,3	**42,0**	3,2	50,0	1,4	0,7	0,4	4,3	16,0	**34,0**	37,8	4,1	0,9	2,9	2,4	5,6	1,5	**87,9**	1,2	1,0	0,4
**Experiment**	**View**	**Resolution**	**Objects**																					**Average Recognition for All Objects**						
			**Ball Valve-S**							**Butterfly Valve**							**3-Way Valve**													
			1	2	3	4	**5**	6	7	1	2	3	4	5	**6**	7	1	2	3	4	5	6	**7**							
FVSR	Full	Same	29,4	3,4	9,4	0,7	**47,8**	0,8	8,6	6,2	3,8	1,3	0,4	4,8	**70,1**	13,6	12,4	1,7	2,1	0,2	4,3	2,4	**76,8**	69,8						
FVDR		Different	6,9	4,9	3,7	65,4	**14,6**	1,5	2,9	4,0	6,6	2,8	49,0	2,6	**22,5**	12,4	4,1	6,3	7,2	55,9	5,2	1,0	**20,4**	29,0						
PVSR	Partial	Same	18,5	14,1	13,0	14,0	**33,1**	1,4	6,0	7,0	4,5	2,7	11,3	5,2	**54,3**	14,9	14,5	3,0	4,0	12,2	8,2	1,6	**56,5**	50,9						
PVDR		Different	6,1	5,8	3,4	66,6	**12,6**	1,9	3,5	5,2	6,1	3,1	51,0	3,0	**20,4**	11,3	5,1	7,3	6,3	57,4	5,7	0,9	**17,2**	24,9						
FVSR/ FVDR	**Average Over**	Full View	18,1	4,2	6,5	33,0	**31,2**	1,2	5,8	5,1	5,2	2,0	24,7	3,7	**46,3**	13,0	8,3	4,0	4,7	28,0	4,7	1,7	**48,6**	49,4						
PVSR/ PVDR		Partial View	12,3	10,0	8,2	40,3	**22,8**	1,6	4,8	6,1	5,3	2,9	31,1	4,1	**37,4**	13,1	9,8	5,2	5,2	34,8	6,9	1,3	**36,8**	37,9						
FVSR/ PVSR		Same Res	23,9	8,8	11,2	7,3	**40,5**	1,1	7,3	6,6	4,2	2,0	5,9	5,0	**62,2**	14,2	13,5	2,3	3,0	6,2	6,3	2,0	**66,6**	60,3						
FVDR/ PVDR		Diff Res	6,5	5,4	3,5	66,0	**13,6**	1,7	3,2	4,6	6,4	2,9	50,0	2,8	**21,5**	11,8	4,6	6,8	6,8	56,6	5,4	0,9	**18,8**	27,0						
**FVSR/** **FVDR/** **PVSR/** **PVDR**	**Full Average**		15,2	7,1	7,4	36,7	**27,0**	1,4	5,3	5,6	5,3	2,5	27,9	3,9	**41,8**	13,0	9,1	4,6	4,9	31,4	5,8	1,5	**42,7**	43,6						

**Table 7:** Average of recognition per resolution for all descriptors using the same resolution between the model and the scan. The results are shown for 6 different noise levels and for 2 cases, full and partial object views.

	Different Standard Deviation (σ)		
	σ **=** **0**	σ **=** **0.00625**	σ **=** **0.0125**
**Full Object View**	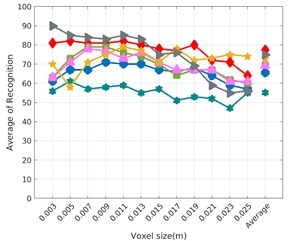	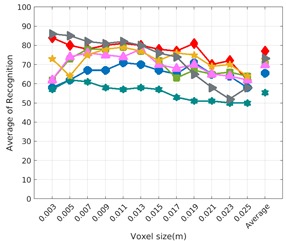	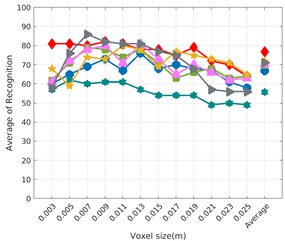
	σ **=** **0.025**	σ **=** **0.05**	σ **=** **0.1**
**Full Object View**	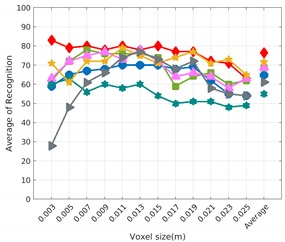	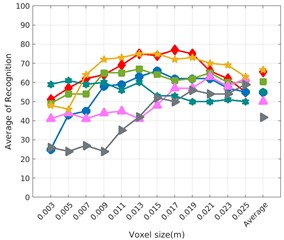	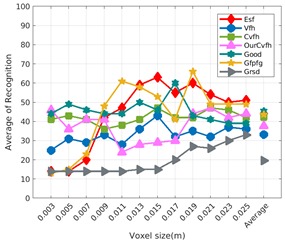
	σ **=** **0**	σ **=** **0.00625**	σ **=** **0.0125**
**Partial Object View**	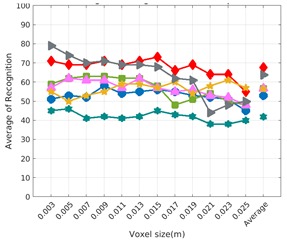	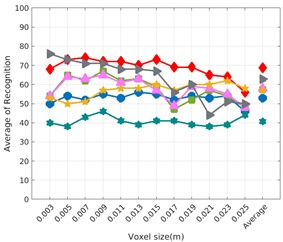	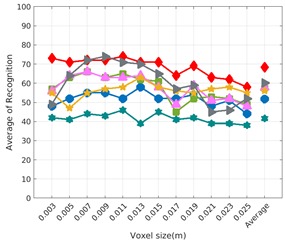
	σ **=** **0.025**	σ **=** **0.05**	σ **=** **0.1**
**Partial Object View**	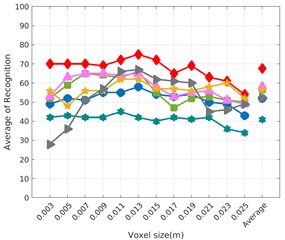	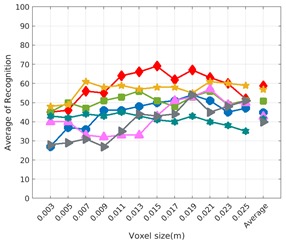	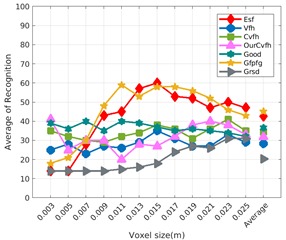

**Table 8:** Average Recognition for all noise levels and all descriptors, averaged by resolution. The two best performing descriptors are marked in green and light green respectively, and the worst performing ones in dark and light red.

Noise Std	Experiment	View	Resolution	Descriptors							Average Over Descriptors
				ESF	VFH	CVFH	OURCVFH	GOOD	GFPFH	GRSD	
σ=0	FVSR	Full	Same	80,7	65,9	77,7	77,0	**61,9**	71,1	**85,6**	74,3
	PVSR	Partial		69,7	52,3	63,9	61,4	**41,7**	53,4	**70,4**	59,0
σ=0.00625	FVSR	Full	Same	78,4	67,4	78,6	76,4	**61,0**	75,4	**82,6**	74,3
	PVSR	Partial		74,6	52,9	62,4	63,6	**43,7**	51,7	**71,7**	60,1
σ=0.0125	FVSR	Full	Same	80,0	69,1	79,6	78,0	**60,3**	74,7	**86,1**	75,4
	PVSR	Partial		72,3	55,3	66,7	66,3	**44,4**	55,9	**72,3**	61,9
σ=0.025	FVSR	Full	Same	**80,4**	67,4	78,3	75,9	**56,4**	72,1	61,4	70,3
	PVSR	Partial		**70,4**	51,4	65,9	65,6	**42,9**	56,7	51,3	57,7
σ=0.05	FVSR	Full	Same	62,4	45,7	54,7	41,9	59,7	**64,6**	**27,7**	51,0
	PVSR	Partial		56,0	36,1	36,1	33,4	44,3	**61,4**	**31,1**	42,7
σ=0.1	FVSR	Full	Same	20,0	29,7	42,6	42,4	**45,7**	23,3	**14,3**	31,1
	PVSR	Partial		28,9	23,7	30,0	30,7	**40,7**	30,3	**14,7**	28,4
Average	FVSR/PVSR	Average	Same	**64,5**	51,4	61,4	59,4	**50,2**	57,6	55,8	57,2

**Table 9:** Average of recognition per different standard deviation for the resolution 0.007 and for all descriptors: (top-left) Using full object views and the same resolution between the model and the measurement; (top-right) Using partial object views and the same resolution between the model and the measurement.

	Full Object View	Partial Object View
**Same Resolution**	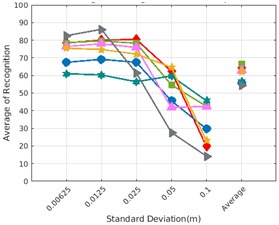	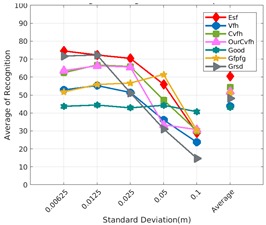

**Table 10:** Confusion table for all objects and noises.

	**Experiment**	**View**	**Resolution**	**Objects**																												
				**Ball Valve**							**Elbow**							**R-Tee**							**R-Socket**							
				**1**	2	3	4	5	6	7	1	**2**	3	4	5	6	7	1	2	**3**	4	5	6	7	1	2	3	**4**	5	6	7	
σ=0	FVSR	Full	Same	**59,9**	2,9	23,6	0,6	13,7	0,4	17,4	0,7	**84,7**	7,0	7,0	0,1	0,4	0,0	3,9	15,3	**71,9**	1,7	3,9	2,0	1,4	0,9	2,3	0,3	**95,9**	0,7	0,0	0,0	
	PVSR	Partial		**45,7**	9,0	7,9	0,4	21,1	0,3	15,6	3,6	**66,0**	5,3	22,6	1,1	0,6	0,9	3,9	31,0	**54,7**	5,1	3,9	0,6	0,9	2,1	6,1	0,9	**88,6**	1,3	0,3	0,7	
	**FVSR/** **PVSR**	**Average**		**52,8**	5,9	15,7	0,5	17,4	0,4	16,5	2,1	**75,4**	6,1	14,8	0,6	0,5	0,4	3,9	23,1	**63,3**	3,4	3,9	1,3	1,1	1,5	4,2	0,6	**92,2**	1,0	0,1	0,4	
σ=0.00625	FVSR	Full	Same	**57,9**	3,0	6,1	0,7	11,9	1,0	19,4	0,4	**81,1**	8,6	8,3	0,1	1,3	0,1	3,4	14,3	**75,7**	2,0	3,0	0,9	0,7	1,4	3,7	0,7	**93,7**	0,4	0,0	0,0	
	PVSR	Partial		**45,6**	9,1	8,7	0,3	21,4	0,3	14,6	1,9	**64,4**	4,9	26,1	0,4	0,9	1,4	4,1	30,3	**55,4**	5,1	3,4	0,9	0,7	2,1	5,0	0,6	**89,3**	2,0	0,4	0,6	
	**FVSR/** **PVSR**	**Average**		**51,7**	6,1	7,4	0,5	16,6	0,6	17,0	1,1	**72,8**	6,7	17,2	0,3	1,1	0,8	3,8	22,3	**65,6**	3,6	3,2	0,9	0,7	1,8	4,4	0,6	**91,5**	1,2	0,2	0,3	
σ=0.125	FVSR	Full	Same	**58,9**	2,7	6,0	0,7	14,6	0,7	16,4	1,0	**85,9**	4,4	7,9	0,1	0,4	0,3	3,4	16,7	**72,9**	2,3	2,6	1,3	0,9	1,3	1,7	0,9	**95,0**	1,1	0,0	0,0	
	PVSR	Partial		**48,9**	10,4	7,6	1,0	17,4	0,7	14,0	2,9	**69,1**	4,6	20,7	1,6	0,4	0,7	5,3	32,1	**54,7**	3,0	2,3	1,3	1,3	2,1	6,3	1,0	**88,3**	1,7	0,3	0,3	
	**FVSR/** **PVSR**	**Average**		**53,9**	6,6	6,8	0,9	16,0	0,7	15,2	1,9	**77,5**	4,5	14,3	0,9	0,4	0,5	4,4	24,4	**63,8**	2,6	2,4	1,3	1,1	1,7	4,0	0,9	**91,6**	1,4	0,1	0,1	
σ=0.025	FVSR	Full	Same	**62,0**	4,3	3,6	0,6	12,9	0,3	16,4	0,3	**79,1**	8,7	11,1	0,1	0,1	0,4	3,0	18,7	**69,0**	1,7	5,1	0,7	1,7	1,4	2,7	1,3	**93,6**	1,0	0,0	0,0	
	PVSR	Partial		**54,3**	7,4	10,0	0,9	15,1	0,0	12,3	2,3	**59,7**	6,7	28,3	1,0	0,6	1,4	4,4	31,6	**53,6**	5,1	3,1	1,0	1,1	3,0	6,1	1,3	**87,0**	2,0	0,4	0,1	
	**FVSR/** **PVSR**	**Average**		**58,1**	5,9	6,8	0,7	14,0	0,1	14,4	1,3	**69,4**	7,7	19,7	0,6	0,4	0,9	3,7	25,1	**61,3**	3,4	4,1	0,9	1,4	2,2	4,4	1,3	**90,3**	1,5	0,2	0,1	
σ=0.05	FVSR	Full	Same	**30,7**	7,9	2,4	0,4	13,1	15,0	30,4	5,9	**42,4**	9,9	26,0	12,7	0,4	2,7	11,4	28,7	**30,7**	3,6	12,3	6,3	7,0	0,7	5,3	0,4	**91,9**	1,7	0,0	0,0	
	PVSR	Partial		**25,4**	12,4	5,2	0,3	21,1	12,8	22,9	5,3	**30,9**	4,0	43,7	13,0	0,6	2,6	11,3	32,9	**25,9**	5,3	14,5	2,6	7,5	2,4	6,7	1,0	**85,9**	3,3	0,7	0,0	
	**FVSR/** **PVSR**	**Average**		**28,0**	10,1	3,8	0,4	17,1	13,9	26,7	5,6	**36,6**	6,9	34,9	12,9	0,5	2,6	11,4	30,8	**28,3**	4,4	13,4	4,4	7,3	1,6	6,0	0,7	**88,9**	2,5	0,4	0,0	
σ=0.1	FVSR	Full	Same	**8,3**	18,7	15,9	0,3	7,3	25,7	23,9	1,1	**27,6**	8,9	27,9	15,0	14,4	5,1	14,1	34,0	**15,3**	3,3	3,3	16,3	13,7	1,3	16,4	4,1	**49,0**	14,0	14,3	0,9	
	PVSR	Partial		**8,1**	25,1	17,7	1,4	4,4	24,7	18,4	0,7	**20,7**	11,7	33,7	13,3	14,9	5,0	15,9	29,1	**17,1**	6,9	7,9	15,6	7,6	1,4	14,7	2,3	**61,3**	5,9	13,9	0,6	
	**FVSR/** **PVSR**	**Average**		**8,2**	21,9	16,8	0,9	5,9	25,2	21,1	0,9	**24,1**	10,3	30,8	14,1	14,6	5,1	15,0	31,6	**16,2**	5,1	5,6	15,9	10,6	1,4	15,6	3,2	**55,1**	9,9	14,1	0,7	
				**Objects**																												
	**Experiment**	**View**	**Resolution**	**Ball Valve-S**							**Butterfly Valve**							**3-Way Valve**							**Average** **Confusion**							**Recogn-ition**
				1	2	3	4	**5**	6	7	1	2	3	4	5	**6**	7	1	2	3	4	5	6	**7**	**1**	**2**	**3**	**4**	**5**	**6**	**7**	
σ=0	FVSR	Full	Same	23,6	5,0	10,4	1,0	**48,3**	0,6	11,1	6,9	7,7	3,0	0,0	5,7	**65,9**	10,9	12,3	2,4	2,9	0,7	3,1	0,7	**77,9**	8,0	5,9	7,9	1,8	4,5	0,7	6,8	**72,0**
	PVSR	Partial		20,6	14,1	10.9	1,1	**42,1**	1,4	9,7	6,9	5,7	4,3	0,6	6,7	**61,4**	14,4	15,6	3,9	4,1	1,0	5,0	0,6	**69,9**	8,8	11,6	5,5	5,1	6,5	0,6	7,0	**61,2**
	**FVSR/** **PVSR**	**Average**		22,1	9,6	10,6	1,1	**45,2**	1,0	10,4	6,9	6,7	3,6	0,3	6,2	**63,6**	12,6	13,9	3,1	3,5	0,9	4,1	0,6	**73,9**	8,4	8,8	6,7	3,5	5,5	0,7	6,9	**66,6**
σ=0.00625	FVSR	Full	Same	19,1	4,6	10,4	1,0	**50,7**	1,7	12,4	6,3	4,1	1,9	0,1	4,3	**69,3**	14,0	9,9	2,1	3,9	0,4	2,1	3,0	**78,6**	6,8	5,3	5,3	2,1	3,6	1,3	7,8	**72,4**
	PVSR	Partial		18,0	11,3	11,1	0,9	**44,7**	2,7	11,3	6,6	5,6	3,9	0,4	7,4	**64,9**	11,3	15,9	2,9	4,3	0,4	6,3	1,1	**69,1**	8,1	10,7	5,6	5,5	6,8	1,0	6,6	**61,9**
	**FVSR/** **PVSR**	**Average**		18,6	7,9	10,8	0,9	**47,7**	2,2	11,9	6,4	4,9	2,9	0,3	5,9	**67,1**	12,6	12,9	2,5	4,1	0,4	4,2	2,1	**73,9**	7,4	8,0	5,4	3,8	5,2	1,2	7,2	**67,2**
σ=0.125	FVSR	Full	Same	27,7	3,6	6,6	0,4	**52,3**	0,4	9,0	7,4	4,0	2,4	0,4	2,9	**73,4**	9,4	12,4	1,7	2,6	0,6	3,3	1,6	**77,9**	8,9	5,1	3,8	2,0	4,1	0,7	6,0	**73,7**
	PVSR	Partial		15,3	9,4	11,9	1,4	**49,1**	1,3	11,6	5,9	3,7	1,1	1,1	6,4	**68,9**	12,9	16,9	3,4	4,6	0,7	7,7	0,9	**65,9**	8,0	10,9	5,1	4,7	6,2	0,8	6,8	**63,6**
	**FVSR/** **PVSR**	**Average**		21,5	6,5	9,2	0,9	**50,7**	0,9	10,3	6,6	3,9	1,8	0,8	4,6	**71,1**	11,1	14,6	2,6	3,6	0,6	5,5	1,2	**71,9**	8,5	8,0	4,5	3,4	5,1	0,8	6,4	**68,6**
σ=0.025	FVSR	Full	Same	35,7	5,3	8,9	0,9	**39,7**	0,3	9,3	11,1	5,6	1,9	0,4	3,7	**62,4**	14,9	13,1	2,0	3,9	0,9	3,7	0,6	**75,9**	10,8	6,4	4,7	2,6	4,4	0,3	7,1	**68,8**
	PVSR	Partial		25,3	12,1	14,0	1,9	**35,9**	1,1	9,7	12,7	5,3	3,3	0,4	6,0	**54,9**	17,4	18,9	3,4	4,0	0,0	4,3	0,3	**69,1**	11,1	11,0	6,5	6,1	5,3	0,6	7,0	**59,2**
	**FVSR/** **PVSR**	**Average**		30,5	8,7	11,4	1,4	**37,8**	0,7	9,5	11,9	5,4	2,6	0,4	4,9	**58,6**	16,1	16,0	2,7	3,9	0,4	4,0	0,4	**72,5**	10,9	8,7	5,6	4,3	4,8	0,5	7,1	**64,0**
σ=0.05	FVSR	Full	Same	20,3	11,0	7,0	2,6	**26,9**	14,1	18,1	4,1	6,7	2,0	0,4	2,6	**61,7**	22,4	8,6	3,6	3,0	0,0	5,1	15,7	**64,0**	8,5	10,5	4,1	5,5	7,9	8,6	13,5	49,8
	PVSR	Partial		18,4	17,2	11,6	1,9	**27,1**	8,6	15,2	5,5	4,5	2,6	0,5	4,4	**52,0**	30,5	9,2	4,4	3,8	0,6	11,4	14,9	**55,7**	8,7	13,0	4,7	8,7	11,3	6,7	13,1	43,3
	**FVSR/** **PVSR**	**Average**		19,3	14,1	9,3	2,3	**27,0**	11,4	16,7	4,8	5,6	2,3	0,5	3,5	**56,9**	26,5	8,9	4,0	3,4	0,3	8,3	15,3	**59,9**	8,6	11,8	4,4	7,1	9,6	7,6	13,3	46,5
σ=0.1	FVSR	Full	Same	11,1	27,9	15,3	0,3	**7,7**	21,3	16,4	1,3	8,1	6,6	1,3	1,0	**50,9**	30,9	3,1	8,0	11,1	0,1	1,4	28,6	**47,6**	5,4	18,9	10,3	5,5	7,0	20,1	15,1	29,5
	PVSR	Partial		13,3	29,3	16,4	1,9	**7,6**	18,6	13,0	0,9	10,0	5,0	1,3	1,3	**49,1**	32,4	3,6	11,4	8,6	0,1	2,4	27,1	**46,7**	6,0	20,0	10,3	7,5	5,9	19,1	12,8	30,1
	**FVSR/** **PVSR**	**Average**		12,2	28,6	15,9	1,1	**7,6**	19,9	14,7	1,1	9,1	5,8	1,3	1,1	**50,0**	31,6	3,4	9,7	9,9	0,1	1,9	27,9	**47,1**	5,7	19,4	10,3	6,5	6,4	19,6	14,0	29,8
																								**Average**	**8,2**	**10,8**	**6,2**	**4,8**	**6,1**	**5,1**	**9,1**	

**Table 11:** Confusion Matrix for the real and synthetic data, for all descriptors, represented in a table.

		Objects																	
Descriptors	Experiment	Ball Valve				Elbow				R-Tee				R-Socket				Average of Recognition	
		1	2	3	4	1	2	3	4	1	2	3	4	1	2	3	4	Real	Synth-etic
**ESF**	Real	**100**	0	0	0	0	**76**	24	0	8	0	**92**	0	21	2	0	**77**	86,2	
	Synthetic	**100**	0	0	0	1	**99**	0	0	0	0	**100**	0	0	0	0	**100**		99,8
**VFH**	Real	**100**	0	0	0	31	**41**	21	7	4	6	**90**	0	19	2	0	**60**	72,8	
	Synthetic	**100**	0	0	0	0	**100**	0	0	0	0	**100**	0	0	**48**	0	**52**		88,0
**CVFH**	Real	**100**	0	0	0	0	**93**	3	3	0	4	**95**	0	0	6	0	**94**	**95,4**	
	Synthetic	**100**	0	0	0	0	**100**	0	0	0	0	**100**	0	0	0	0	**100**		**100**
**OURCVFH**	Real	**100**	0	0	0	0	**83**	0	17	0	0	**96**	4	0	13	0	**88**	91,5	
	Synthetic	**100**	0	0	0	0	**100**	0	0	0	0	**100**	0	0	0	0	**100**		**100**
**GOOD**	Real	**100**	0	0	0	34	**66**	0	0	100	0	**0**	0	33	0	0	**67**	58,0	
	Synthetic	**100**	0	0	0	0	**100**	0	0	**100**	0	**0**	0	0	0	0	**100**		**75,0**
**GFPFH**	Real	**60**	0	0	40	0	**48**	0	52	4	73	**23**	0	0	15	0	**85**	**54,1**	
	Synthetic	**100**	0	0	0	0	**88**	**12**	0	1	0	**99**	0	0	0	0	**100**		96,8
**GRSD**	Real	**88**	0	12	0	0	**76**	0	24	0	33	**67**	0	0	2	0	**98**	82,1	
	Synthetic	**100**	0	0	0	0	**98**	2	0	0	0	**100**	0	0	0	0	**100**		99,5
**Average Real**		**92,6**	0,0	1,7	5,7	9,3	69,0	6,9	14,8	16,7	16,6	65,9	0,6	10,4	5,6	0,0	81,2		

**Table 12:** Example of the performance of the two descriptors ESF and GFPFH: (First column) scanned view with the corresponding model view and the correct view in case of mis-recognition; (Second column) visualisation of the corresponding histograms for each descriptor.

	Scanned/(Miss-)Recognized/Correct View	Histogram of the Descriptor
**ESF**	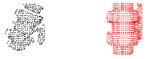	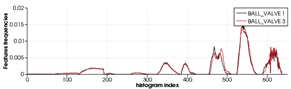
**GFPFH**	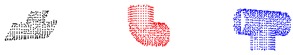	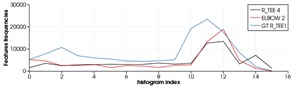

## Abbreviations

The following abbreviations are used in this manuscript:

MP	Motion Primitive
BoF	Bag-of-Features
CVFH	Clustered Viewpoint Feature Histogram
CSHOT	Colored Signature of Histogram of Orientation
CAD	Computer aided design model
CRF	Conditional Random Field model
ECSAD	Equivalent circumference surface angle descriptor
EFPFH	Extended Fast Point Feature Histograms
FPFH	Fast Point Feature Histograms
GFPFH	Global Fast Point Feature Histogram
GOOD	Global Orthographic Object Descriptor
GRSD	Global Radius-based Surface Descriptors
GPCSD	Global Principal Curvature Shape Descriptor
IMR	Inspection Maintenance and repair
RSD	Radius-based Surface Descriptors
OUR-CVFH	Oriented, Unique and Repeatable
PVC	Polyvinylchloride pressure pipes
PCL	Point Cloud Library
PCA	Principal Component Analysis
RCS	Rotational Contour Signatures
RoPS	Rotational Projection Statistics
ROVs	Remotely Operated Vehicles
SDC	Shape Distribution Component
SLAM	Simultaneous Localization And Mapping
SHOT	Signature of Histogram of Orientation
SI	Spin Image
VFH	Viewpoint Feature Histogram
FVSR	Full View Same Resolution Experiment
PVSR	Partial View Same Resolution Experiment
FVDR	Full View Different Resolution Experiment
PVDR	Partial View Different Resolution Experiment
NFVSR	Noisy Full View Same Resolution Experiment
NPVSR	Noisy Partial View Same Resolution Experiment
IK	Inverse Kinematics
UVMS	Underwater Vehicle Manipulator System
I-AUV	Intervention Autonomous Underwater Vehicle
AUV	Autonomous Underwater Vehicle
ROV	Remotely Operated Vehicle
DoF	Degrees of Freedom
DVL	Doppler Velocity Log
FOV	Field Of View
LIDAR	Light Detection and Ranging
